# Nanomolar Inhibitors of the Transcription Factor STAT5b with High Selectivity over STAT5a**

**DOI:** 10.1002/anie.201410672

**Published:** 2015-02-20

**Authors:** Nagarajan Elumalai, Angela Berg, Kalaiselvi Natarajan, Andrej Scharow, Thorsten Berg

**Affiliations:** Institute of Organic Chemistry, University of LeipzigJohannisallee 29, 04103 Leipzig (Germany)

**Keywords:** inhibitors, natural products, protein–protein interactions, STAT5, transcription factors

## Abstract

Src homology 2 (SH2) domains play a central role in signal transduction. Although many SH2 domains have been validated as drug targets, their structural similarity makes development of specific inhibitors difficult. The cancer-relevant transcription factors STAT5a and STAT5b are particularly challenging small-molecule targets because their SH2 domains are 93 % identical on the amino acid level. Here we present the natural product-inspired development of the low-nanomolar inhibitor Stafib-1, as the first small molecule which inhibits the STAT5b SH2 domain (*K*_i_=44 nm) with more than 50-fold selectivity over STAT5a. The binding site of the core moiety of Stafib-1 was validated by functional analysis of point mutants. A prodrug of Stafib-1 was shown to inhibit STAT5b with high selectivity over STAT5a in tumor cells. Stafib-1 provides the first demonstration that naturally occurring SH2 domains with more than 90 % sequence identity can be selectively targeted with small organic molecules.

Src homology 2 (SH2) domains are highly homologous regions, approximately 100 amino acids long, which are found in a significant subset of signaling proteins. SH2 domains play a fundamental role in intracellular signaling via recognition of specific phosphotyrosine-containing peptide motifs.[1] The conserved nature and similar binding preferences of these domains pose an enormous challenge for specific inhibitor development.[2] A particularly challenging example is given by the SH2 domains of the transcription factors STAT5a and STAT5b,[3] which are essential for STAT5a/b signaling. These are 93 % identical on the amino acid level (see Figure S1 in the Supporting Information), and bind to the same peptide motifs.[4] Consequently, STAT5a and STAT5b are often jointly referred to as “STAT5”, despite tissue-specific expression patterns and a number of non-redundant biological functions.[3,5] Antisense oligonucleotides directed against STAT5b, but not STAT5a, inhibited tumor growth in mice.[6] Most recently, selective inhibition of Stat5b has been proposed as a novel therapeutic approach to combat Bcr-Abl positive leukemias.[7] Small-molecule inhibitors targeting the SH2 domain of STAT5b with specificity over the STAT5a SH2 domain would be useful for investigating the differential roles of STAT5a and STAT5b under diverse conditions, and could serve as lead compounds for drug development.

While a small number of STAT5 SH2 domain inhibitors have been reported,[8] none of these studies has disclosed selectivity for one STAT5 protein over the other. We recently reported Fosfosal as a STAT5b SH2 domain inhibitor (*K*_i_=17.4 μm)[9] (Figure 1 a). Structure–activity relationships of Fosfosal revealed that deleting the carboxylic acid retained partial activity, but deleting the phosphate led to a complete loss of activity against STAT5b. In an attempt to increase activity, we substituted the carboxylic acid group of Fosfosal with a second phosphate. This structural change is contrary to standard medicinal chemistry, which aims to replace phosphate groups with isosteric groups of lesser charge. However, the resulting compound, catechol bisphosphate (**1**), inhibited STAT5b with an inhibitory constant *K*_i_ of only 0.9±0.1 μm (Figures 1 a,b, Table 1, and see Tables S1 and S2 in the Supporting Information). Surprisingly, analysis of **1** against STAT5a in a newly developed binding assay (see Figure S2 and Supporting Methods in the Supporting Information) revealed 35-fold lower activity against the SH2 domain of STAT5a (*K*_i_=34±3 μm). The SH2 domains of other STAT family members and the tyrosine kinase Lck[10] were only inhibited at higher concentrations. Positioning the two phosphate groups of **1** in the *meta* position, as represented by resorcinol bisphosphate (**2**), resulted in a strong decrease of activity against STAT5b (*K*_i_=27±4 μm, Table 1), with concomitant loss of selectivity. Replacement of the two phosphate groups by methylene diphosphonates, as in the compound **3**, resulted in virtually complete loss of activity against STAT5b (Table 1 and Table S1).

**Figure 1:**
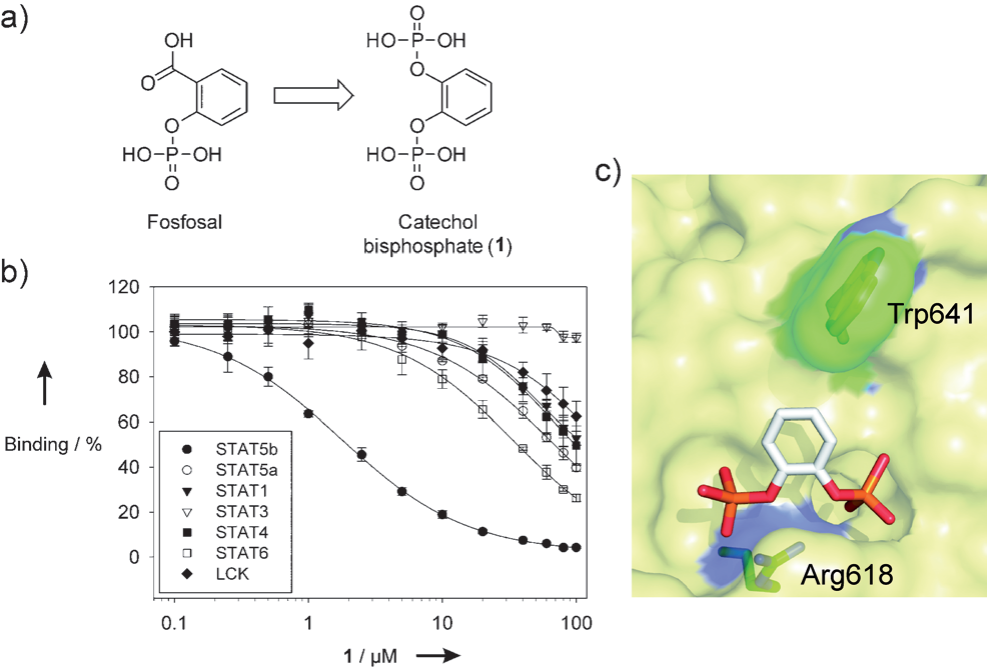
Catechol bisphosphate (1) is a selective inhibitor of the STAT5b SH2 domain. a) Structures of Fosfosal[9] and 1. b) Activities of 1 against the SH2 domains of STAT family members and the tyrosine kinase Lck analyzed in binding assays based on fluorescence polarization. c) Binding of 1 to the STAT5b SH2 domain as predicted by docking with AutoDock Vina.

**Table 1:** Structures and activities of inhibitors against STAT5b and STAT5a.

Compounds	*K*_i_ [μm] (STAT5b)	*K*_i_ [μm] (STAT5a)	Selec- tivity factor	
**1**	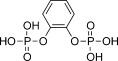	0.93± 0.07	34±3	37
				
**2**	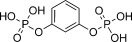	27±4	24±3	0.9
				
**3**	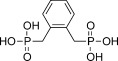	n.a.	n.a.	n.a.
				
**4**	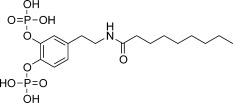	0.69± 0.04	19.2± 2.7	28
				
**5**	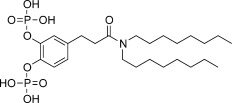	0.82± 0.05	2.5± 0.2	3
				
**6**	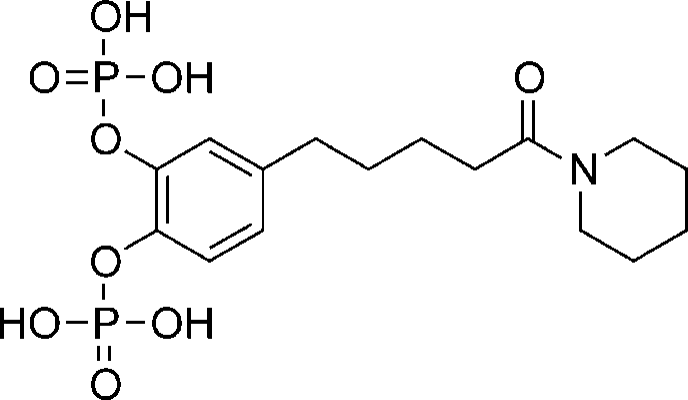	0.73± 0.07	21±5	30
				
**7**	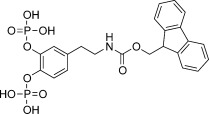	0.45± 0.04	16.0± 0.8	35
				
**8**	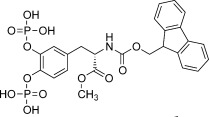	0.46± 0.08	7.8± 1.0	17
				
**9**	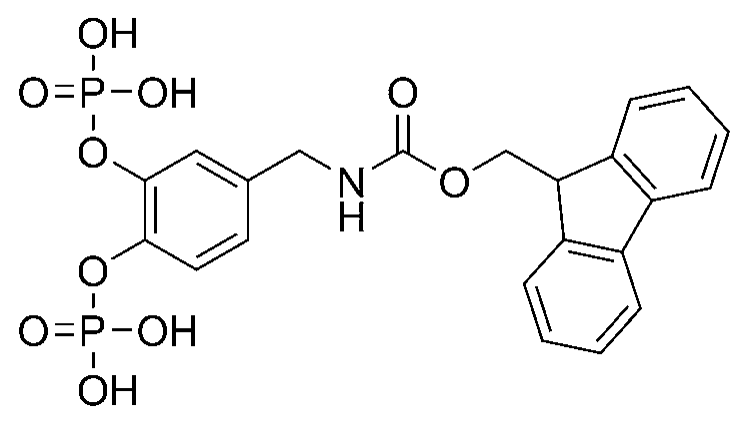	0.21± 0.04	11±2	52
				
**10**	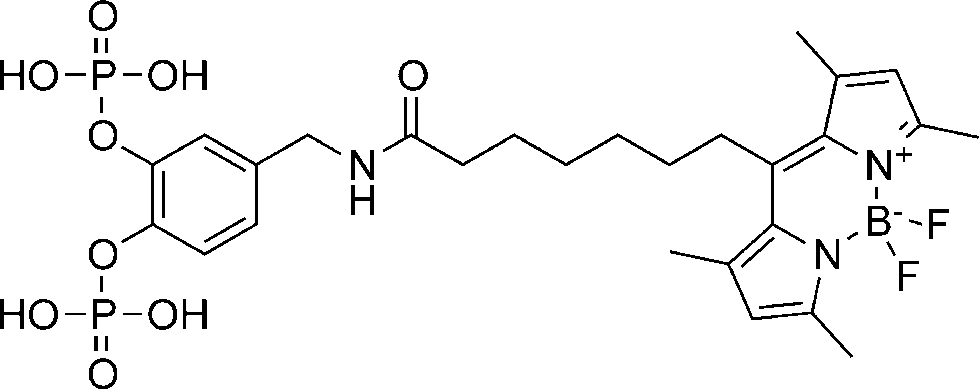	n.a.	n.a.	n.a.
				
**11**	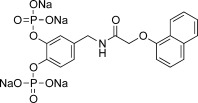	0.24± 0.01	16.1± 1.1	67
				
**12**	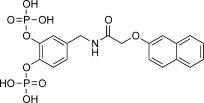	0.28± 0.02	9.3± 0.3	33
				
**13**	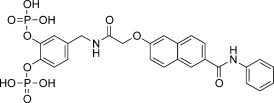	0.044± 0.001	2.42± 0.05	55
				
**14**	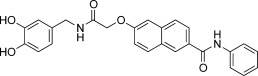	n.a.	n.a.	n.a.
				
**15**	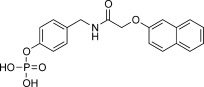	50±3	40±2	0.80
				
**16**	QDTpYLVLDKWL	0.54± 0.08	0.41± 0.09	0.75

The selectivity factor was calculated as *K*_i_ (STAT5a)/*K*_i_ (STAT5b). Conversion of IC_50_ values into *K*_i_ values was carried out as described.[15] n.a.=not applicable.

We carried out molecular docking studies using AutoDock Vina[11] and the previously described homology model of the STAT5b SH2 domain[9] based on the crystal structure of STAT5a.[12] The top three docking results placed catechol bisphosphate into the phosphotyrosine binding pocket of the STAT5b SH2 domain, consistent with electrostatic interactions between STAT5b Arg618 and both phosphate groups (Figure 1 c). The arginine residue in position 618 of STAT5b is highly conserved in all SH2 domains and forms essential electrostatic interactions with the phosphotyrosine moiety of its natural interaction partners.[13] Alignment of the STAT5b homology model[9] with the X-ray structure of STAT5a[12] suggested that small differences in the primary structure of STAT5a and STAT5b might cause a partial change in secondary structure in the vicinity of the binding pocket for **1** (see Figure S3 in the Supporting Information). Detailed comparative structural analysis by crystallographic or NMR-based methods would be required to identify the subtle structural differences contributing to specificity.

In attempts to further increase the submicromolar affinity of **1** for STAT5b, we sought inspiration from natural products with an explicit or masked 1,2-dihydroxyphenyl motif such as dopamine, hydrocaffeic acid, piperine, and L-DOPA (see Scheme S1 in the Supporting Information). Since our synthetic methodology for bisphosphate generation involved hydrogenolysis of dibenzylphosphate esters,[14] any double bonds in the natural products are saturated in our derivatives. The N-acylated dopamine derivative **4** (*K*_i_=0.69±0.04 µm), the hydrocaffeic acid derivative **5** (*K*_i_=0.82±0.05 µm), and the tetrahydropiperine derivative **6** (*K*_i_=0.73±0.07 µm) displayed similar or marginally higher activities than **1** (Table 1 and Table S1). However, the Fmoc-protected dopamine bisphosphate **7** (*K*_i_=0.45±0.04 µm) and Fmoc-protected L-Dopa methyl ester bisphosphate **8** (*K*_i_=0.46±0.08 µm) are approximately twice as active as **1**. Docking studies suggested π-stacking interactions with Trp641 as a potential cause, and suggested that shifting the amide bond of **7** closer to the catechol moiety might facilitate hydrogen bonds between the inhibitor and the protein backbone at amino acid positions 642 and 644 (see Figure S4 in the Supporting Information). Indeed, **9** (*K*_i_=0.21±0.04 µm) is twofold more active than **7**. All catechol bisphosphate derivatives, except **5**, display strong selectivity for STAT5b over STAT5a (Table 1 and Table S1).

Before further compound optimization, we verified the binding site of **1** (Figure 1 c). The BODIPY-FL-labeled derivative **10** (see Scheme S2 in the Supporting Information) was designed as a tracer molecule for direct binding assays based on fluorescence polarization, and was found to bind to wild-type STAT5b with high affinity (*K*_d_=0.86±0.08 μm; Figure 2). Consistent with the binding mode proposed by docking (Figure 1 c), binding of **10** to the STAT5b point mutant Arg618Ala was markedly reduced as compared to Stat5b wild-type. Binding to STAT5b Arg618Lys, with a positive charge one carbon–carbon bond closer to the protein backbone than in wild-type STAT5b, was partially reduced. Binding to STAT5b Trp641Ala was significantly reduced, suggesting that Trp641 is important for binding of the catechol bisphosphate core. The weak affinity of wild-type STAT5a for **10** is in line with the results of the competition-based fluorescence polarization assay (Figure 1 b and Table 1). These results indicate that the catechol bisphosphate binds to the phosphotyrosine binding pocket of the STAT5b SH2 domain, confirming its selectivity for STAT5b over STAT5a.

**Figure 2:**
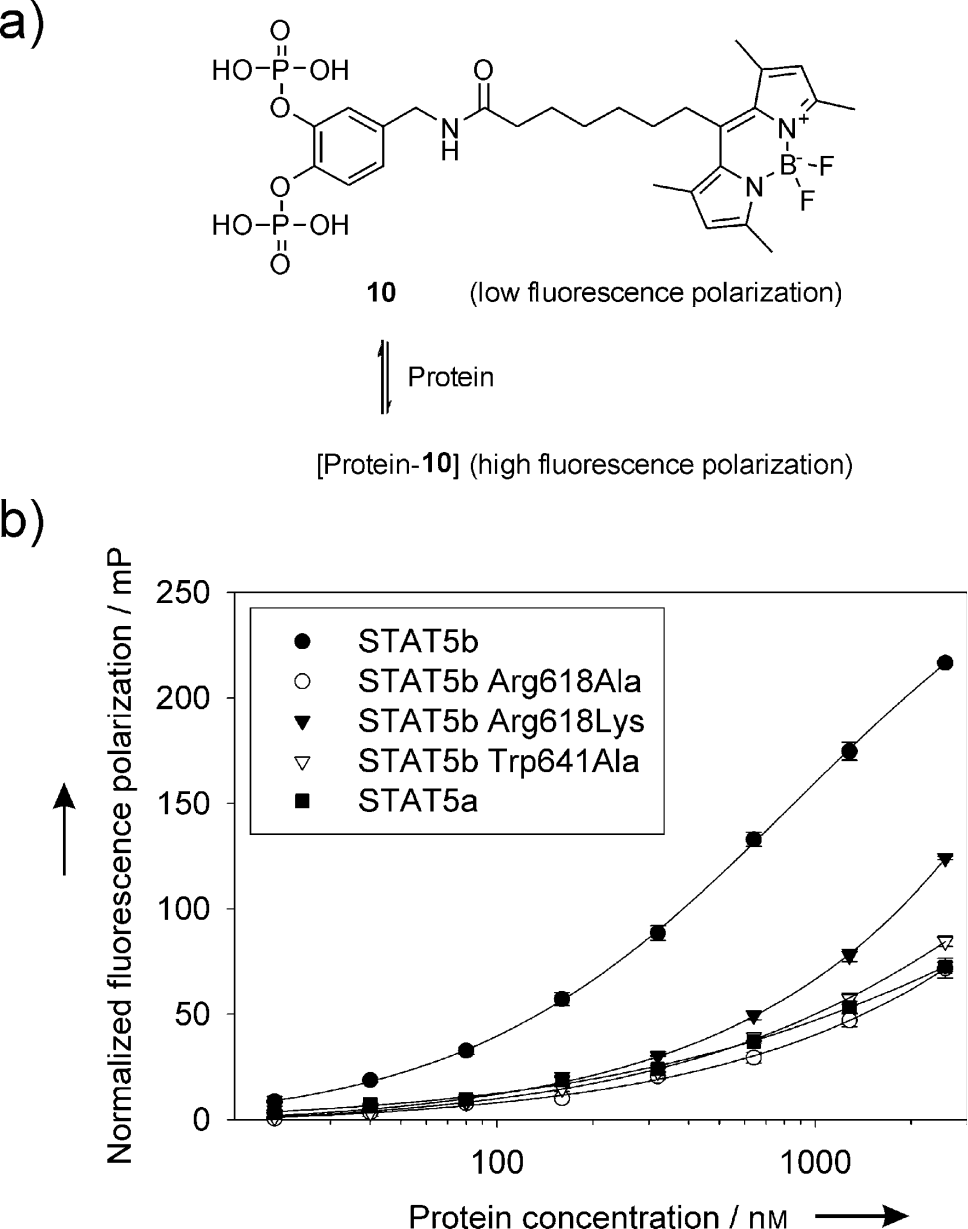
Validation of the binding mode and specificity of catechol bisphosphate derivatives. a) Structure of the BODIPY-FL-labeled catechol bisphosphate derivative 10 and principle of the assay. b) 10 nm of 10 were incubated with the indicated wild-type and mutant STAT5 proteins at the indicated protein concentrations. Binding was detected by an increase in fluorescence polarization. *K*_d_ values for the STAT5b mutants and STAT5a could not be determined, since their extrapolated values exceed the highest protein concentration tested (2560 nm).

Replacement of the Fmoc group of **9** with a naphthyl group resulted in the compounds **11** (*K*_i_=0.24±0.01 µm) and **12** (*K*_i_=0.28±0.02 µm; Table 1). In order to further improve the activity of the compounds, we envisioned targeting the hydrophobic pocket created by Phe633 and Tyr665 by an aromatic group. We designed compound **13** by extending the core of **12** via an *N*-phenyl carboxamide group (Figure 3 a,b) for synthesis in an eight-step procedure (Figure 3 c). Compound **12** was chosen over **11** as a template, since it displays more favorable geometry for extending to the adjacent hydrophobic pocket. Compound **13** displayed fourfold higher activity against STAT5b (*K*_i_=0.044±0.001 μm) than **12** (Figure 3 d, Table 1), and displays 55-fold selectivity for STAT5b over STAT5a (*K*_i_ (STAT5a)=2.42±0.05 μm; see Table S3 in the Supporting Information), and even higher selectivity against other SH2 domains. Removal of both phosphate groups as in **14** led to a complete loss of activity. Monophosphorylated **15**, which is based on **12**, was also significantly less active, and lost specificity for STAT5b. These data, as well as data from additional control compounds, demonstrate the fundamental importance of the bisphoshorylated core structure for selective STAT5b inhibition (see Table S4 in the Supporting Information).

**Figure 3:**
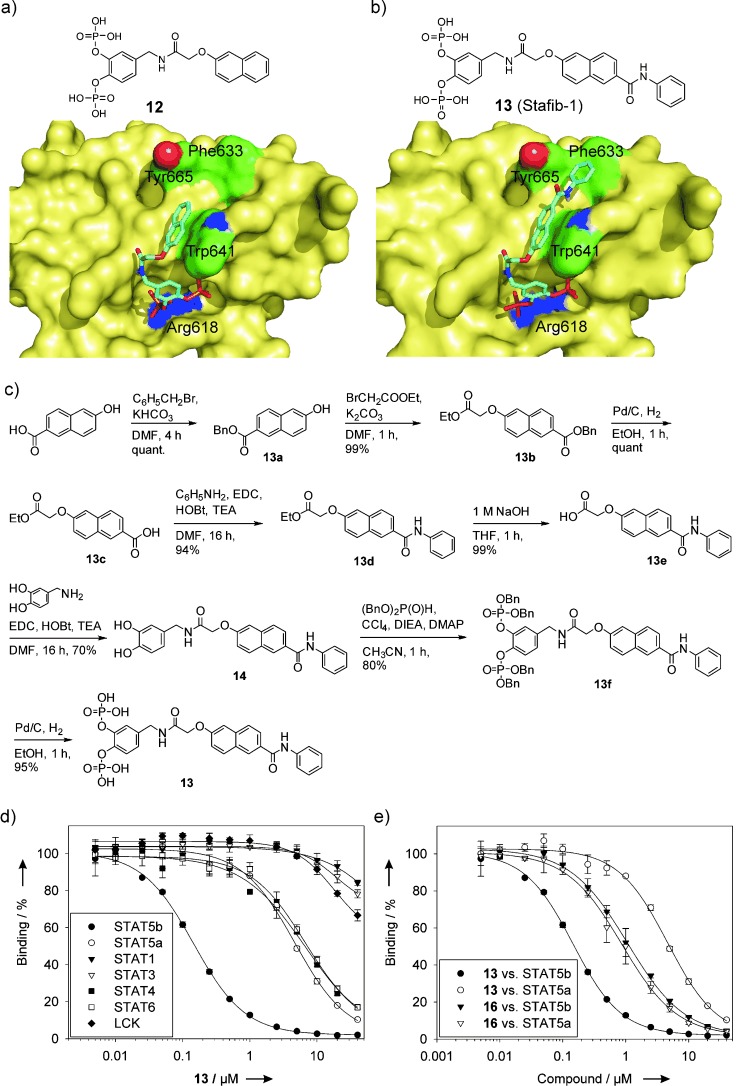
Design, synthesis, and biochemical activity of compound 13. a) Docking of 12 into the STAT5b SH2 domain with AutoDock Vina. b) Binding mode of 13 as suggested by docking. c) Synthesis of 13. d) Activities of 13 against the SH2 domains of STAT family members and the tyrosine kinase Lck. e) Comparison of the activities of 13 and the natural peptide ligand QDTpYLVLDKWL (16) against STAT5a and STAT5b.

The high activity and selectivity of **13** prompted us to carry out a comparison with the natural ligand QDTpYLVLDKWL (**16**), derived from the EPO receptor.[16] The fluorescence polarization assays we used for STAT5a and STAT5b are both based on STAT5a/b binding to the core sequence of this peptide motif, pYLVLDKWL.[17] Both STAT5a (*K*_d_=133±26 nm) and STAT5b (*K*_d_=103±13 nm) bind to 5-carboxylfluorescein-pYLVLDKWL with similar affinities. The inhibitory constants of the peptide QDTpYLVLDKWL (**16**) against the STAT5 proteins were also similar (*K*_i_ (STAT5a): 0.41±0.09 μm, *K*_i_ (STAT5b)=0.54±0.08 μm), demonstrating that the natural peptide sequence does not differentiate between the two STAT5 proteins (Figure 3 e). In contrast, **13** discriminates between STAT5a and STAT5b, binding to STAT5b with higher affinity and ligand efficiency (LE) than the native EPO-receptor-derived peptide [LE (**13**)=0.18; LE (**16**)=0.07].

A key event in STAT signaling is STAT binding to activated cell surface receptors and/or non-receptor tyrosine kinases (Figure 4 a).[18] Binding is required for STAT phosphorylation at the conserved tyrosine residue C-terminal of their SH2 domain (STAT5a: Tyr694; STAT5b: Tyr699). The efficiency of STAT SH2 domain inhibitors can thus be investigated by analyzing the phosphorylation state of STAT proteins, using phospho-specific antibodies for the conserved tyrosine. However, analysis of endogenous Stat5a and Stat5b phosphorylation is hampered by the lack of antibodies able to selectively recognize tyrosine-phosphorylated STAT5b but not tyrosine-phosphorylated STAT5a (or vice versa). All current commercial antibodies recognizing phosphorylated STAT5b (pTyr699) and/or STAT5a (pTyr694) have been raised against a phosphotyrosine-containing peptide which is identical in both proteins. In order to analyze the relative activity of small molecules against the individual STAT5 proteins, we transfected K562 cells with expression vectors for the fusion proteins STAT5a-GFP or STAT5b-GFP. Like endogenous STAT5a/b, the fusion proteins are phosphorylated at the conserved tyrosine residue. However, the presence of the GFP tag increases the molecular weight, allowing the fusion proteins to be distinguished from endogenous Stat5 on a Western blot.

**Figure 4:**
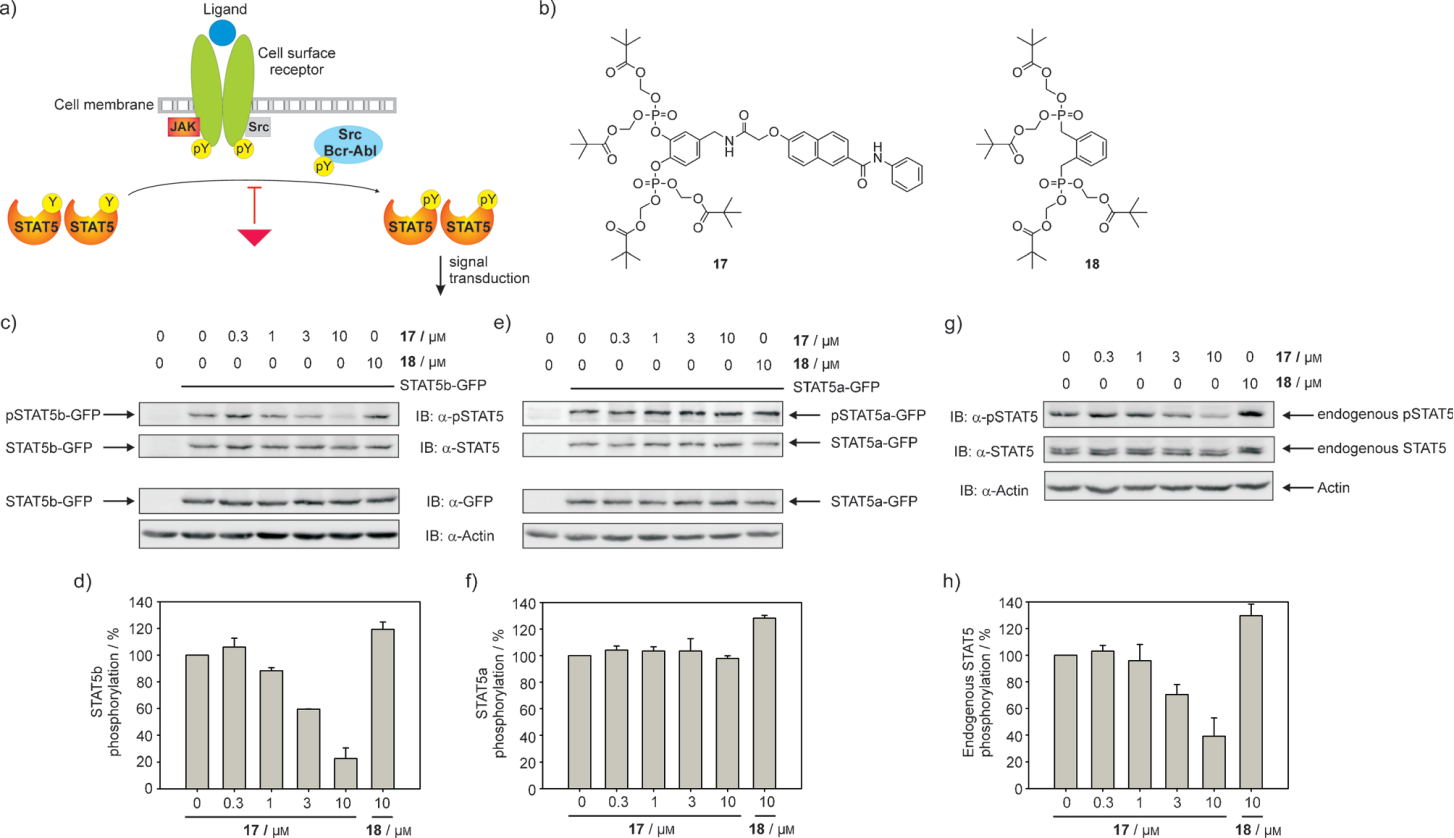
Selective inhibition of STAT5b tyrosine phosphorylation by 17, the pivaloyloxymethyl ester of 13. a) STAT signaling is induced by activated cell surface receptors and non-receptor tyrosine kinases. A small-molecule inhibitor of a STAT SH2 domain (the red triangle) inhibits STAT signaling by inhibiting STAT phosphorylation at the conserved tyrosine residue. The graphic is modified from the literature.[20] b) Structure of 17 and the negative control compound 18. c) Inhibition of STAT5b tyrosine phosphorylation by 17 in K562 cells transfected with STAT5b-GFP. A separate gel was run to confirm even transfection via the GFP tag. d) Quantification of the pSTAT5b-GFP bands, normalized against total STAT5b-GFP. Error bars represent the standard deviations from two independent experiments. e) Tyrosine phosphorylation of STAT5a in K562 cells transfected with STAT5a-GFP is not inhibited by 17. A separate gel was run to confirm even transfection via the GFP tag. f) Quantification of the pSTAT5a-GFP bands, normalized against total STAT5a-GFP. Error bars represent the standard deviations from two independent experiments. g) Effect of 17 and 18 on tyrosine phosphorylation of endogenous STAT5. h) Quantification of the endogenous pSTAT5 bands, normalized against total endogenous STAT5. Error bars represent the standard deviations from four independent experiments.

To test its activity in cell-based assays, **13** was converted into the corresponding cell-permeable pivaloyloxymethyl (POM) ester **17**, which is designed to liberate **13** inside the cell after cleavage by intracellular esterases (Figure 4 b).[19] To rule out the possibility that inhibitory effects of **17** on STAT5b phosphorylation might be caused by the release of pivalic acid or formaldehyde generated during the cleavage, the pivaloyloxymethylester **18**, based on the inactive bisphosphonate **3**, was prepared as a control compound. Neither **17** nor **18** displayed activity against STAT5b in vitro (see Table S1). Treatment of Stat5b-GFP-transfected K562 cells with **17** showed a dose-dependent decrease of Tyr699 phosphorylation of STAT5b-GFP (Figure 4 c,d). Timecourse experiments indicated a steady increase in inhibition during the first hour of exposure to **17**, consistent with cleavage of the POM groups to liberate the active agent, and inhibition was still observed after 8 hours (see Figure S5 in the Supporting Information). The negative control compound **18** did not inhibit phosphorylation. Treatment of Stat5a-GFP-transfected K562 cells with **17** did not affect phosphorylation of Tyr694 of STAT5a-GFP (Figure 4 e,f).

These data demonstrate that the high specificity of **13** observed in vitro is also maintained under cellular conditions. Since endogenous STAT5 consists of both STAT5a and STAT5b, the effect of **17** on endogenous STAT5 (Figure 4 g,h) is lower than the effect on STAT5b-GFP (Figure 4 c,d). Based on the relative degrees of inhibition of endogenous STAT5 and STAT5b-GFP, we conclude that the majority of phosphorylated endogenous STAT5 in K562 cells is STAT5b. Studies quantifying the relative amounts of STAT5a and STAT5b and their phosphorylation levels in K562 cells have not been published.

In summary, we have developed **13** as the first small molecule that can differentiate between the two highly homologous STAT5 proteins. **13**, dubbed Stafib-1 (for “*Sta*t *fi*ve *b* inhibitor-1”), displays low-nanomolar activity against STAT5b (*K*_i_=44±1 nm), with more than 50-fold selectivity over STAT5a. This is the first report describing strongly divergent affinities of the SH2 domains of STAT5a/b for a chemical entity, both in vitro and in cultured human cells. The binding site of the core of the inhibitors, catechol bisphosphate (**1**), on STAT5b was characterized by point mutants, which represents the first point mutant validation of a small-molecule inhibitor of a STAT SH2 domain. The pivaloyloxymethylester of Stafib-1, dubbed Pomstafib-1 (**17**), inhibited tyrosine phosphorylation of a STAT5b-GFP fusion protein with high selectivity over the corresponding STAT5a-GFP fusion protein in human leukemia cells. **17** will be a valuable tool for dissecting the functions of STAT5b and STAT5a in mammalian cells. To the best of our knowledge, our work represents the first demonstration that small molecules can differentiate between naturally occurring SH2 domains which have more than 90 % amino acid sequence identity. We believe that our data will significantly influence the scientific community’s perception of the potential of small molecules as potent and selective inhibitors of highly similar protein–protein interaction domains.
