# Supplementary material for: Nanomolar Inhibitors of the Transcription Factor STAT5b with High Selectivity over STAT5a
Source: Angew Chem Int Ed Engl. 2015 Feb 20;54(16):4758–63. doi: 10.1002/anie.201410672 (PMC4471549; doi:10.1002/anie.201410672)
Supplement: Supplementary file 1 [file anie0054-4758-sd1.pdf]

Angewandte Chemie
Eine Zeitschrift der Gesellschaft Deutscher Chemiker

Supporting Information

Nanomolar Inhibitors of the Transcription Factor STAT5b with High Selectivity over STAT5a**

*Nagarajan Elumalai, Angela Berg, Kalaiselvi Natarajan, Andrej Scharow, and Thorsten Berg**

anie_201410672_sm_miscellaneous_information.pdf

Table of contents

Supporting Figures and Tables 2
Figure S1 2
Figure S2 2
Figure S3 3
Figure S4 4
Figure S5 4
Scheme S1 5
Scheme S2 5
Table S1 6
Table S2 7
Table S3 8
Table S4 8
Supporting methods 9
Plasmid construction and protein expression 9
Fluorescence polarization assays 9
Molecular docking 9
Tissue culture experiments 10
General synthetic methods 10
Synthesis and spectroscopic characterization of compounds 11
Supporting references 33

Supporting Figures and Tables

Figure S1

STAT5a SH2 domain (amino acids 594-684)

```
ILGFVNKQQAHDLLINKPDGTFLLRFSDSEIGGITIAWKFDSPERNLWNLKPFTTRDFSIRSLADRLGDLSYLIYVFPDRPKDEVFSKYYT
ILGFVNKQQAHDLLINKPDGTFLLRFSDSEIGGITIAWKFDSQERMFWNLMPFTTRDFSIRSLADRLGDLNYLIYVFPDRPKDEVYSKYYT
```

STAT5b SH2 domain (amino acids 594-684)

Figure S1. Sequence comparison of the SH2 domains of human STAT5a (upper line, NCBI reference NM_003152ORF) and human STAT5b (lower line, NCBI reference NM_012448). 6 of the 91 amino acids are different; these are highlighed in red and underlined.

Figure S2

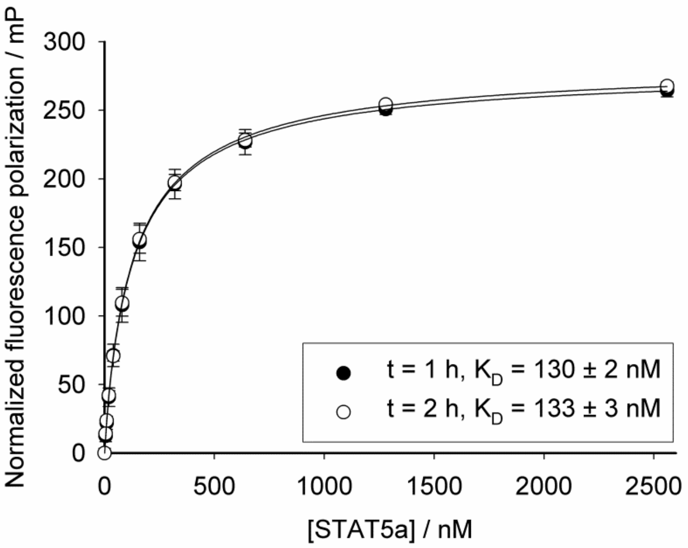

Figure S2. Binding curve for STAT5a in a fluorescence polarization assay. STAT5a was incubated at room temperature in assay buffer at the indicated concentrations for 1 h to mimic the incubation period with test compounds. Subsequently, the fluorophore-labeled peptide 5-carboxyfluorescein-GY(PO_3H_2)LVLDKW was added (final concentration: 10 nM), and fluorescence polarization measured immediately (t = 1 h, solid circles) or after an additional hour (t = 2 h, empty circles). Both curves are essentially identical, indicating temporal stability of the assay. Fluorescence polarization data are normalized to the values of the fluorophore-labeled peptide in the absence of protein. For details, see the Supporting Methods.

Figure S3

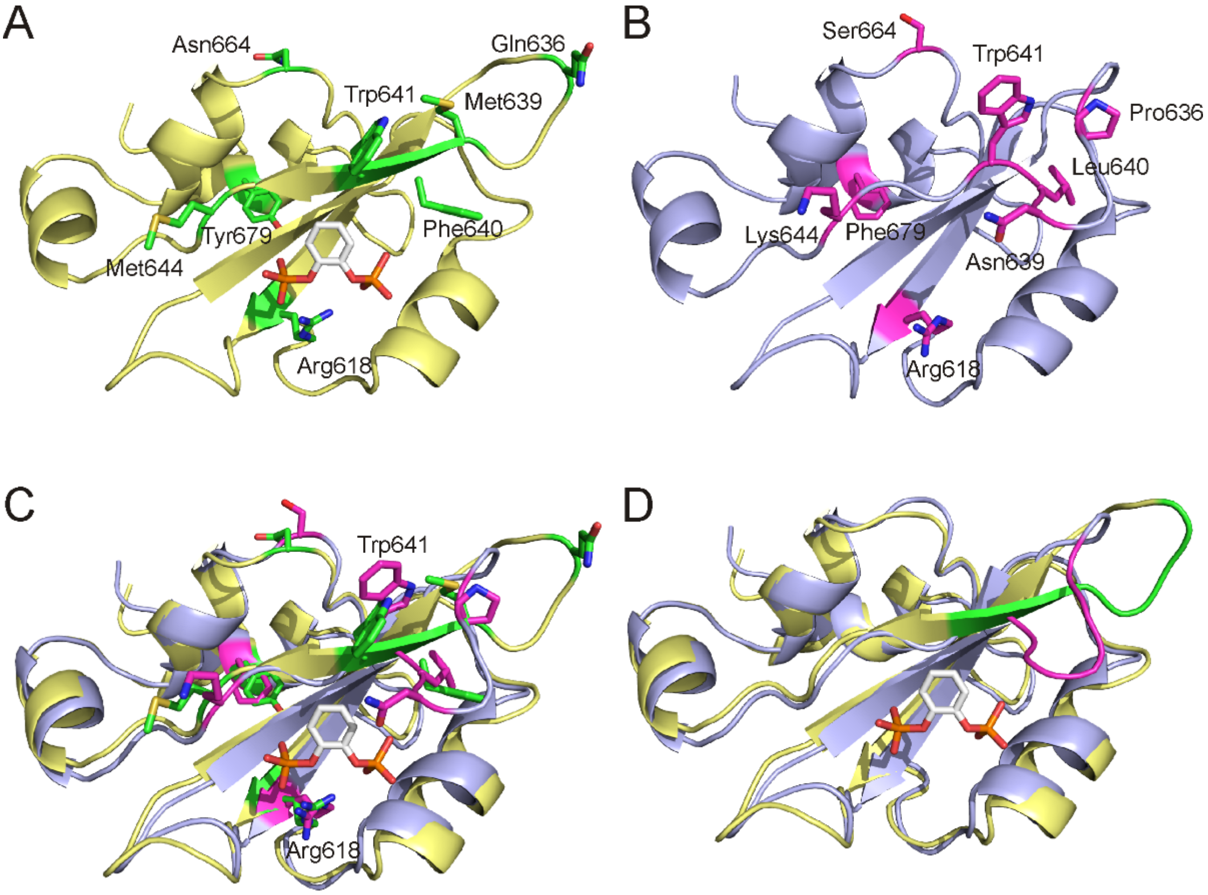

Figure S3. Putative binding of **1** to the homology model of the Stat5b SH2 domain based on the X-ray structure of the Stat5a SH2 domain. A) Model of **1** bound to the STAT5b SH2 domain. The side chains of amino acids divergent in STAT5a and STAT5b at positions 636, 639, 640, 644, 664, and 679 are shown. The side chains of Arg618 and Trp641, which are identical in both STAT5 proteins, are also shown to indicate the location of the binding pocket for **1**. B) STAT5a SH2 domain as reported in the X-ray structure (PDB 1Y1U)[1]. The side chains of amino acids at positions 636, 639, 640, 644, 664, and 679 are shown, together with the side chains of Arg618 and Trp641. C) Overlay of A) and B). STAT5b is depicted in yellow, STAT5a in lightblue. The numbering of the divergent amino acids was omitted for clarity of presentation. D) Overlay of A) and B) as shown in C), but amino acid side chains and numberings are omitted for clarity of depiction. Amino acids positions 636-640, which diverge in secondary structure, are highlighted (STAT5b: green; STAT5a: magenta).

Figure S4

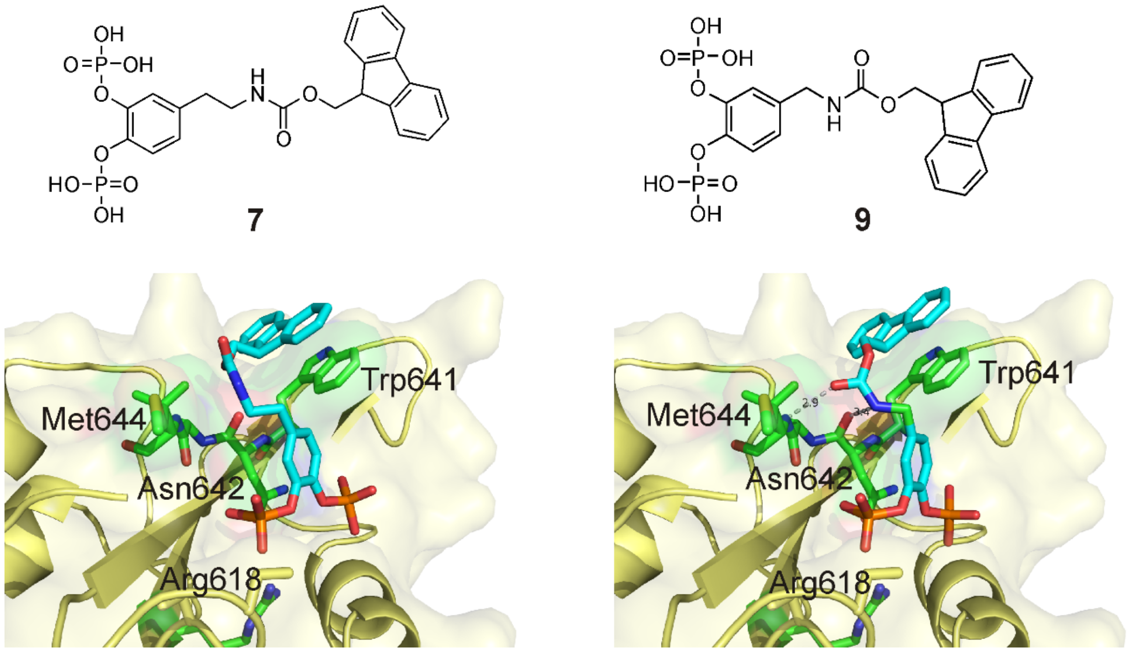

Figure S4. Docking of compounds **7** and **9** into the STAT5b SH2 domain. The amide bond of compound **9** is directed towards the protein, suggesting the possibility of hydrogen bonding with the protein backbone at positions 642 and 644. In contrast, the amide bond of **7** is orientated parallel to the protein backbone, which make hydrogen bonds less likely.

Figure S5

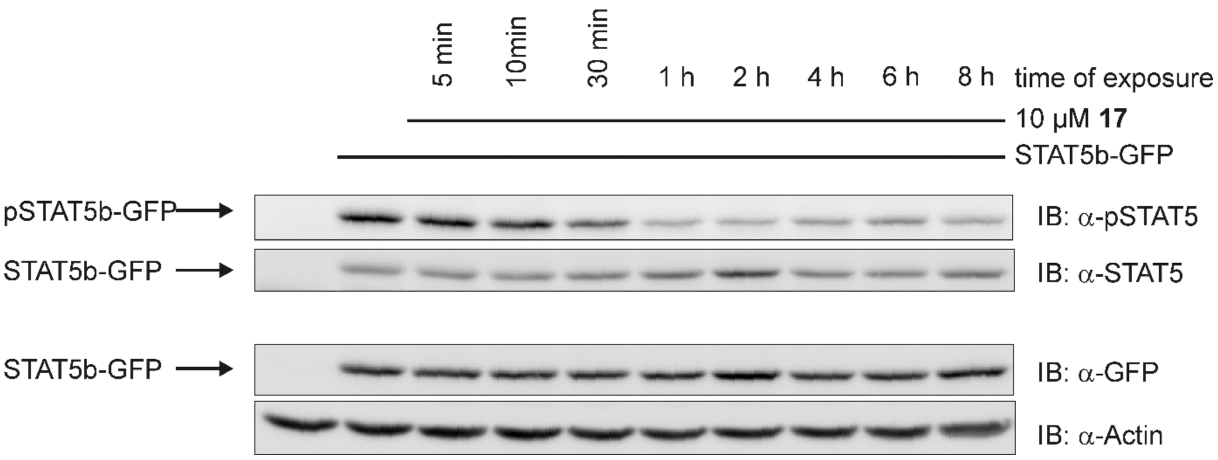

Figure S5. Time course of STAT5b inhibition in K562 cells. K562 cells transfected with STAT5b-GFP were exposed with compound **17** at a concentration of 10 μM for the indicated periods of time. Inhibition of STAT5b tyrosine phosphorylation by **17** was analyzed by Western Blot. A separate gel was run to monitor even transfection via the GFP tag.

Scheme S1

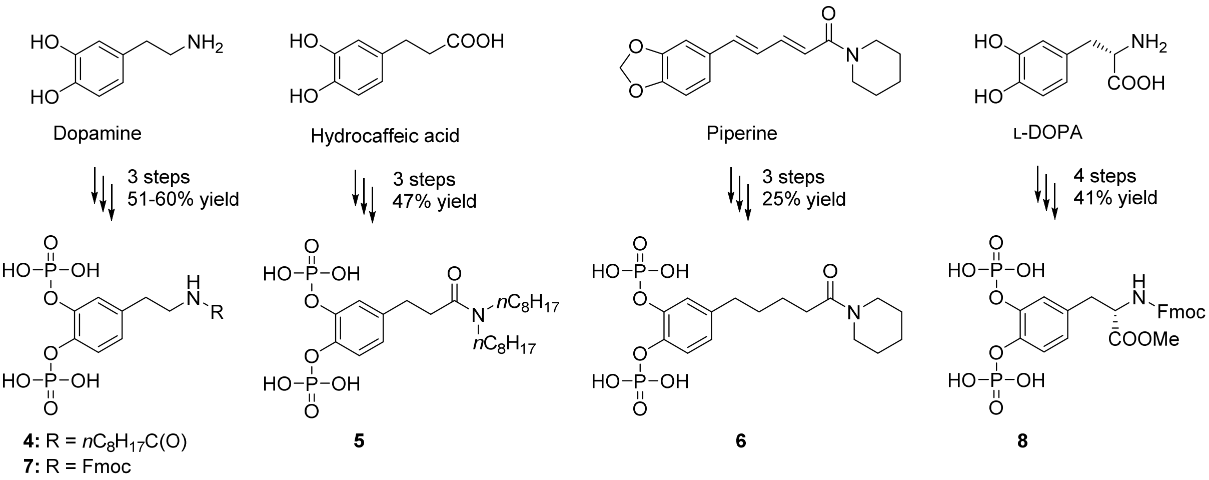

Scheme S1. Natural-product inspired synthesis of catechol bisphosphate derivatives **4-8**.

Scheme S2

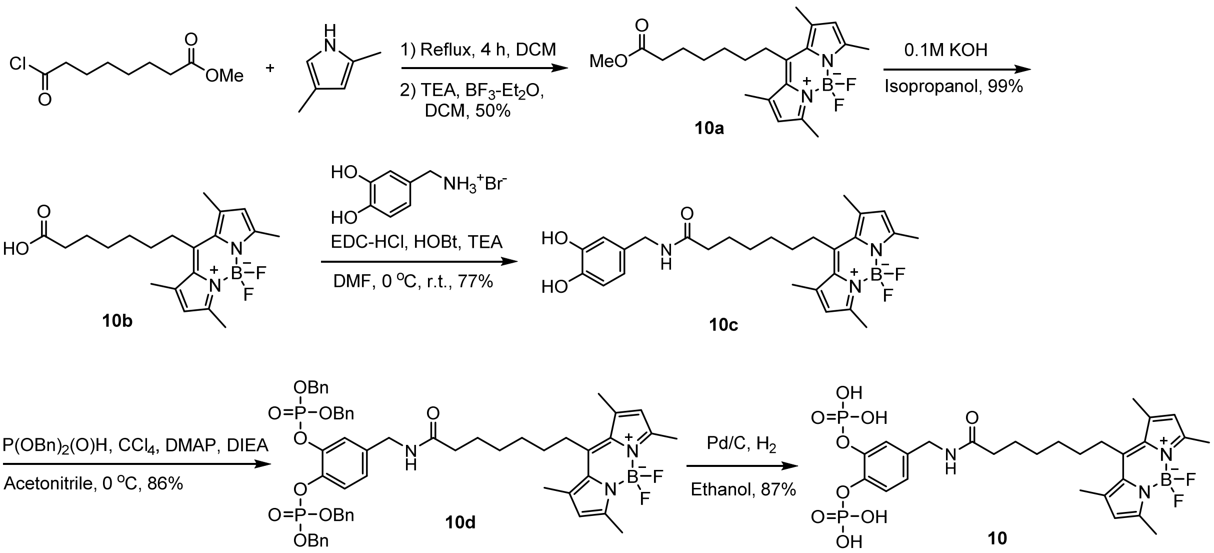

Scheme S2. Synthesis of the BODIPY-FL-labeled catechol bisphosphate derivative **10**.

Table S1

No	Structure	IC_{50} (µM) (STAT5b) or maximum inhibition (%)	K_i (µM) (STAT5b)[a]	IC_{50} (µM) (STAT5a) or maximum inhibition (%)	K_i (µM) (STAT5a)[a]
1	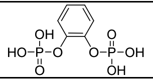	1.95 ± 0.15	0.93 ± 0.07	69 ± 5	34 ± 3
2	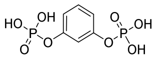	55 ± 10	27 ± 4	50 ± 6	24 ± 3
3	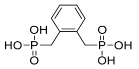	6 ± 2% inhibition at 100 µM	n/a	16 ± 1% inhibition at 100 µM	n/a
4	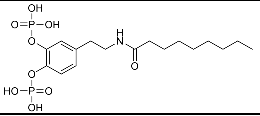	1.44 ± 0.17	0.69 ± 0.04	38.9 ± 5.4	19.2 ± 2.7
5	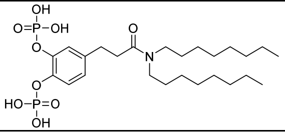	1.64 ± 0.06	0.82 ± 0.05	5.1 ± 0.4	2.5 ± 0.2
6	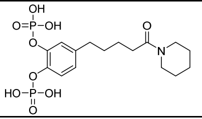	1.53 ± 0.09	0.73 ± 0.07	42 ± 10	21 ± 5
7	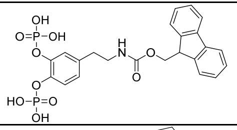	0.97 ± 0.08	0.45 ± 0.04	32.4 ± 1.5	16.0 ± 0.8
8	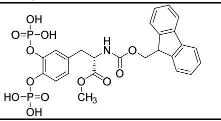	0.98 ± 0.15	0.46 ± 0.08	15.8 ± 2.1	7.8 ±1.0
9	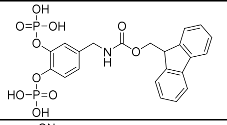	0.46 ± 0.06	0.21 ± 0.04	22.3 ± 3.1	11 ± 2
11	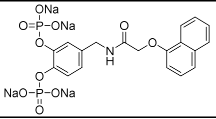	0.55 ± 0.06	0.24 ± 0.01	32.5 ± 2.2	16.1 ± 1.1
12	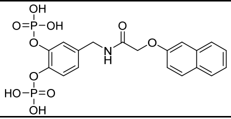	0.63 ± 0.03	0.28 ± 0.02	18.8 ± 0.7	9.3 ± 0.3
13	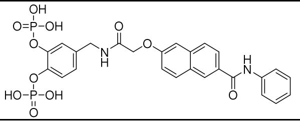	0.154 ± 0.001	0.044 ± 0.001	4.97 ± 0.10	2.42 ± 0.05
14	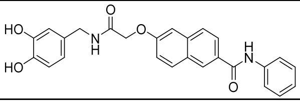	-2 ± 1 % inhibition at 100 µM	n/a	1 ± 1 % inhibition at 100 µM	n/a
15	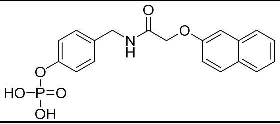	102 ± 6	50 ± 3	81 ± 4	40 ± 2
16	QDTpYLVLDKWL	1.16 ± 0.16	0.54 ± 0.08	0.92 ± 0.18	0.41 ± 0.09

No	Structure	IC_{50} (μM) (STAT5b) or maximum inhibition (%)	K_i (μM) (STAT5b)[a]	IC_{50} (μM) (STAT5a) or maximum inhibition (%)	K_i (μM) (STAT5a)[a]
17	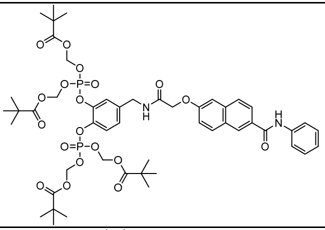	-7 ± 4% inhibition at 100 μM	n/a	n/d	n/d
18	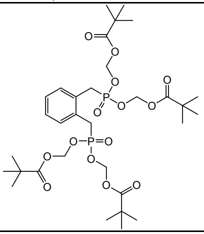	-1 ± 4% inhibition at 100 μM	n/a	n/d	n/d

[a] n/a = not applicable; n/d: not determined.

Table S1. Structures of compounds **1-18** and their activities against STAT5b and STAT5a.

Table S2

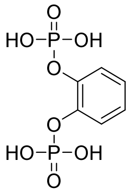

Catechol bisphosphate (1)

Protein	IC_{50} (μM) or maximum inhibition (%)	K_i (μM)
STAT5b	1.95 ± 0.15	0.93 ± 0.07
STAT5a	69 ± 5	34 ± 3
STAT1	90 ± 3	44 ± 1
STAT3	3 ± 2% inhibition at 100 μM	n/a
STAT4	68 ± 6	34 ± 3
STAT6	36 ± 2	18 ± 1
Lck SH2	38 ± 7 % inhibition at 100 μM	n/a

n/a: not applicable

Table S2. Activities of catechol bisphosphate (**1**) in binding assays based on fluorescence polarization.

Table S3

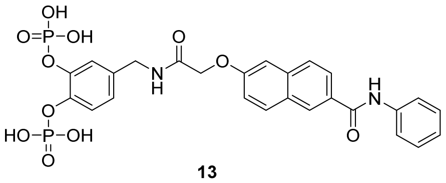

13

Protein	IC_{50} (µM) or maximum inhibition (%)	K_i (µM)
STAT5b	0.154 ± 0.001	0.044 ± 0.001
STAT5a	4.97 ± 0.10	2.42 ± 0.05
STAT1	16 ± 1 % inhibition at 40 µM	n/a
STAT3	22 ± 2 % inhibition at 40 µM	n/a
STAT4	6.58 ± 0.30	3.29 ± 0.15
STAT6	7.17 ± 0.87	3.47 ± 0.43
Lck SH2	33 ± 3 % inhibition at 40 µM	n/a

n/a: not applicable

Table S3. Activities of Stafib-1 (**13**) in binding assays based on fluorescence polarization.

Table S4

No	Structure	Maximum inhibition (%) at 100 µM (STAT5b)	Maximum inhibition (%) at 100 µM (STAT5a)	Source of compound
SI-1	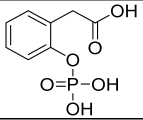	39 ± 4	40 ± 3	Synthesized as part of this work
SI-2	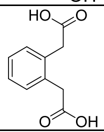	-3 ± 1	-3 ± 4	Sigma 131407
SI-3	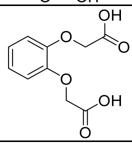	-3 ± 1	-5 ± 5	Alfa Aesar L01843
SI-4	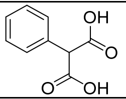	-9 ± 12	-0.4 ± 0.1	Alfa Aesar A16937
SI-5	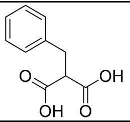	5 ± 14	-10 ± 3	Sigma 100773

Table S4. Activities of control compounds expected to display three (SI-1) or two (SI-2 to SI-5) negative charges at neutral pH, against STAT5b and STAT5a as analyzed in fluorescence polarization assays.

Supporting methods

Plasmid construction and protein expression

Cloning and expression, and purification protocols for STAT1, STAT3, STAT4, STAT5b, STAT6 and the Lck SH2 domain, have been described previously.[2] STAT5a amino acids 137-707 were amplified via PCR and cloned into a modified pQE70 vector carrying a C-terminal 6xHis-tag and an N-terminal maltose-binding protein (MBP) tag. Proteins were expressed from Rosetta BL21DE3 (Novagen) as previously published, and purified via the His-tag on Ni–ion exchange resin. After dialysis against a buffer containing 100 mM NaCl, 50 mM Hepes (pH 7.5), 1 mM EDTA, 1 mM dithiothreitol, 10% glycerol, and 0.1% Nonidet P40, protein concentration was determined via a BCA protein assay (Pierce) and aliquots were snap-frozen in liquid nitrogen and stored at -80 ºC until use.

Fluorescence polarization assays

For competition-based fluorescence polarization assays, the ability of the test compounds to displace fluorophore-labeled peptides from their respective binding proteins was analyzed. Peptide sequences were as follows: STAT1: 5-carboxyfluorescein-GY(PO_3H_2)DKPHVL; STAT3: 5-carboxyfluorescein-GY(PO_3H_2)LPQTV-NH_2; STAT4: 5-carboxyfluorescein-GY(PO_3H_2)LPQNID-OH; STAT5a and STAT5b: 5-carboxyfluorescein-GY(PO_3H_2)LVLDKW; STAT6: 5-carboxyfluorescein-GY(PO_3H_2)VPWQDLI-OH; Lck SH2: 5-carboxyfluorescein-GY(PO_3H_2)EEIP. STAT2 was not analyzed due to protein instability. The final concentration of 5-carboxyfluorescein-labeled peptides used in the FP assays was 10 nM. The final concentration of protein was: STAT1: 420 nM; STAT3: 270 nM; STAT4: 130 nM; STAT5a: 130 nM; STAT5b: 100 nM; STAT6: 310 nM; Lck SH2: 30 nM. These protein concentrations correspond to the dissociation constants (K_d-values) for the individual protein-peptide interactions in assay buffer. Inhibition curves were determined by incubating protein with test compounds for 60 min, followed by addition of the fluorophore-labeled peptides. Fluorescence polarization was analyzed after another 60 min using an Infinite F500 plate reader (Tecan). Stocks of fluorophore-labeled peptides or compound **10**, and protein were diluted in assay buffer, which contains 10 mM Tris (pH 8.0), 50 mM NaCl, 1 mM EDTA, 1 mM DTT, 0.1 % Nonidet P-40 substitute, and 2 % DMSO in water. Assays were performed in black 384-well microtiter plates (Corning no. 3573). Percent inhibition was calculated based on curve fits using SigmaPlot (SPSS Science Software). IC_{50} data were converted to K_i-values using the published equation.[3] For the direct binding assays shown in Figure 2, the BODIPY-FL-labeled catechol bisphosphate derivative **10** was incubated with the indicated proteins for 60 min before fluorescence polarization was analyzed. Changes in the fluorescence polarization values of **10** in the presence of protein are given as normalized fluorescence polarization values.

Ligand efficiencies (LE) were calculated using the following equation[4]

$LE = (-\log_{10} K_i / N)$

where K_i is the inhibitory constant (in mol/L) and N is the number of non-hydrogen atoms.

Molecular docking

The homology model of human STAT5b based on the amino acid sequence of human STAT5a has been described previously.[2] Docking experiments were performed with AutoDock Vina.[5]

Default settings were used with the exception of the exhaustiveness, which was set to 50. The docking poses calculated by AutoDock Vina were visualized using PyMOL.

Tissue culture experiments

The full-length human STAT5a and STAT5b ORFs were PCR-amplified from K562 cDNA, and cloned into an expression vector based on pCS2 carrying a C-terminal GFP tag. K562 cells were cultured in RPMI 1640 medium (Invitrogen), containing 10% FBS (Gibco Life Technologies), 2 mM L-glutamine (PAA Laboratories) and penicillin/streptomycin (PAA Laboratories). Cells were transfected with either STAT5a-GFP or STAT5b-GFP plasmid using Fugene HD Transfection Reagent (Promega; 1 x 10^6 cells per well in 1 ml medium with a 4:1 ratio of Fugene:DNA). After 24 h, the transfected cells were treated with test compound or DMSO for 4 h (final DMSO concentration 0.2 %). Cells were subsequently harvested, and lysed with lysis buffer (50 mM Tris-HCl pH 7.5, 150 mM NaCl, 10 mM $Na_4P_2O_7$, 10% glycerol, 1% Triton X-100, 1 mM EDTA plus protease/phosphatase inhibitors 100 ng/ml aprotinin, 1 m Na_3PO_4, 10 mM NaF and 1 mM PMSF). The components of the cell lysates were separated by SDS-PAGE (10% polyacrylamide gel), then transferred to a nitrocellulose membrane using a semi-dry transfer apparatus. For each experiment, two gels and membranes were prepared. One membrane was probed with Stat5 antibodies (anti-pSTAT5, which recognizes Stat5a pTyr694 and STAT5b pTyr699, Cell Signaling; anti-total Stat5, Cell Signaling; anti-β-actin, Cell Signaling), the other membrane with anti-GFP (Cell Signaling, followed by anti-β-actin) to confirm even transfection. All primary antibodies are rabbit monoclonal antibodies. Membranes with bound primary antibody were incubated with α-rabbit-HRP secondary antibody (Dako) and ECL was performed using Western Lightning Plus chemiluminescence reagent (Perkin-Elmer). Bands were visualized using an ImageQuant digital imaging system (GE Healthcare). Quantitative analysis was performed using ImageJ software (NIH).[6]

General synthetic methods

Method 1: Benzyl phosphorylation of dihydroxy phenyl derivatives

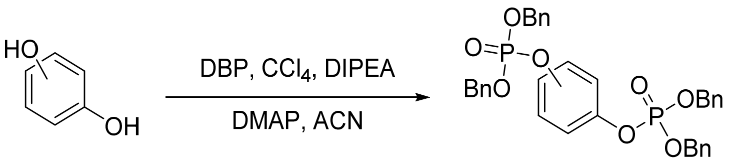

To a stirred solution of aromatic diol (1 mmol) in dry acetonitrile (8 mL) was added CCl_4 (10 mmol), diisopropyl ethylamine (DIPEA) (4 mmol) and catalytic amounts of 4-(dimethylamino)pyridine (DMAP). Dibenzyl phosphite (DBP) (3 mmol) was added dropwise at 0 °C and maintained at these conditions for 0.5-1 h. Upon completion of the reaction, 3 mL of 0.5M KH_2PO_4 were added. The reaction mixture was repeatedly extracted with ethyl acetate, and the combined organic phases were subsequently washed with 5% NaCl solution and H_2O, and were dried over Na_2SO_4. The volatiles were removed under reduced pressure and purified by flash column chromatography.[7]

Method 2: Debenzylation / Hydrogenolysis

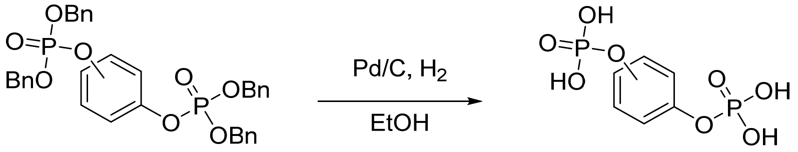

To a solution of benzyl-protected bisphosphate (1 mmol) in absolute ethanol (20 mL) was added 10% Pd/C (20-50 mg) under argon. The argon atmosphere was exchanged for a hydrogen atmosphere. After completion of the reaction (usually 1-2 h, reversed-phase TLC control), the mixture is filtered through celite and washed with ethanol. After removal of the solvent under reduced pressure, the product was dissolved in water and washed with dichloromethane (DCM) (2 x 5 mL). The product was isolated by lyophilization of the aqueous phase.

Method 3: Amide coupling reaction

To a solution of carboxylic acid (1 mmol) in anhydrous DMF (10 mL) was added *N*-(3-dimethylaminopropyl)-*N*′-ethylcarbodiimide hydrochloride (EDC-HCl) (1mmol) and 1-hydroxy benzotriazole hydrate (HOBt) (1 mmol) at 0 °C. The resulting suspension or clear solution was stirred for 30 min at 0 °C and for 1.5 h at room temperature. Triethylamine (3 mmol) and the corresponding primary/secondary amine (1 mmol) were added subsequently, and the mixture was stirred overnight at room temperature. After addition of water (100 mL), the mixture was extracted with ethyl acetate (2 x 30 mL). The combined organic phases were washed with saturated aqueous $NaHCO_3$ solution, and dried over Na_2SO_4. After removal of the solvent under reduced pressure, the product was purified by flash column chromatography.

Method 4: Fmoc protection

The amine or amine hydrochloride (1 mmol) was dissolved in 2 mL of water. 2 mL of saturated aqueous $NaHCO_3$ was added at 0 °C. After 15 min, 9-fluorenyl-methoxycarbonyl chloride (Fmoc-Cl) (1.2 mmol in 3 mL CH_3CN) was added and a white precipitate was formed. The reaction mixture was stirred at 0 °C for 1.5 h, and was then allowed to warm to room temperature. After 16 h, 10 mL of water and 5 mL of 1 M HCl were added. The mixture was extracted with ethyl acetate (3 x 20 ml). The combined organic phases were dried over Na_2SO_4 and concentrated *in vacuo*. The product was purified by column chromatography.

Synthesis and spectroscopic characterization of compounds

1,2-Phenylene tetrabenzyl bis (phosphate) (**1a**)

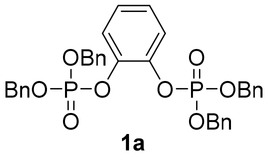

1a

1a was synthesized from catechol (200 mg, 1.8 mmol) according to Method 1**.** The crude product was purified by column chromatography in hexane / ethyl acetate (4:1 → 3:1) to afford

pure product as colorless oil (1.03 g, 90%).[7] R_f = 0.33 (hexane / ethyl acetate 3:1); ^{1}H NMR (400 MHz, $CDCl_3$) δ=5.09 – 5.13 (m, 8H), 7.09 – 7.11 (m, 2H), 7.23 – 7. 38 (m, 22H) ppm; ^{13}C NMR (101 MHz, $CDCl_3$) δ=70.21 (d, *J*=5.9 Hz), 121.79, 125.87, 128.08, 128.65, 128.68, 135.48 (d, *J*=7.4 Hz), 141.06 (t, *J*=6.6 Hz) ppm; ^{31}P NMR (162 MHz, $CDCl_3$) δ= -5.24 (s) ppm; MS (ESI) $C_{34}H_{33}O_8P_2$ calcd: 631.2 [M+H$^+$], found 631.2.

1,2-Phenylene bis(dihydrogen phosphate) (**1**)

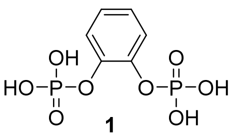

1

1 was synthesized from **1a** (350 mg, 0.55 mmol) according to Method 2 as thick oil (120 mg, 80%).[8] ^{1}H NMR (400 MHz, DMSO-d_6) δ=7.10 – 7.12 (m, 2H), 7.34 (s, 2H), 8.24 (bs, OH) ppm; ^{13}C NMR (101 MHz, DMSO-d_6) δ =122.17, 124.54, 142.69 ppm; ^{31}P NMR (162 MHz, DMSO-d_6) δ= - 4.71 (s); UV/Vis: λ (nm) = 264, 210; IR (Film): $\tilde{\nu}$ = 3407, 2954, 2926, 2855, 2309, 1656, 1641, 1631, 1592, 1507, 1456, 1425, 1270, 1199, 1120, 1029, 978, 907, 638, 597, 510 , 487, 471, 459, 451, 404 cm^{-1}; HRMS (ESI) $C_6H_7O_8P_2$ calcd. 268.9622 [M-H$^+$], found: 268.9623.

1,3-Phenylene tetrabenzyl bis(phosphate) (**2a**)

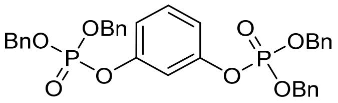

2a

2a was synthesized from resorcinol (200 mg, 1.8 mmol) according to Method 1. The crude product was purified by column chromatography in hexane / ethyl acetate (4:1 → 3:1) and was obtained as colorless oil. Yield: 960 mg (86%). R_f = 0.33 (hexane / ethyl acetate 3:1); ^{1}H NMR (400 MHz, $CDCl_3$) δ=5.09 – 5.11 (m, 8H), 7.00 – 7.10 (m, 2H), 7.19 – 7.21 (m, 1H), 7.33 – 7.32 (m, 21H) ppm; ^{13}C NMR (101 MHz, $CDCl_3$) δ=70.21 (*J*=5.9), 112.71, 116.83 (d, *J*=4.4), 128.17, 128.73, 128.80, 130.38, 135.39 (d, *J*=7.3), 151.23 (d, *J*=6.6); ppm; ^{31}P NMR (162 MHz, $CDCl_3$) δ= -5.46 (s) ppm. MS (ESI) $C_{34}H_{32}NaO_8P_2$ calcd. 653.2 [M+Na$^+$], found: 653.2.

1,3-Phenylene bis(dihydrogen phosphate) (**2**)

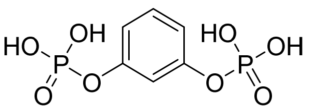

2

2 was synthesized from **2a** (370 mg, 0.59 mmol) according to Method 2 and was obtained as an off-white powder (158 mg, 95 %).[9] Melting point: 165-168 °C; ^{1}H NMR (300 MHz, DMSO-d_6) δ = 6.93 – 6.96 (m, 3H), 7.28 (t, *J*=7.4, 1H), 9.10 (bs, OH); ^{13}C NMR (75 MHz, DMSO-d_6) δ = 152.37 (d, *J*=6.1), 129.83, 115.60 (d, *J*=4.4), 112.45 ppm; ^{31}P NMR (162 MHz, DMSO-d_6) δ = - 5.17 (s) ppm; UV/Vis: λ(nm) = 265, 210; IR (KBr): $\tilde{\nu}$ = 3648, 3537, 3435, 2920, 2852, 2778, 2343, 2290, 1636, 1608, 1483, 1457, 1307, 1274, 1182, 1162, 1146, 1018, 906, 875,

812, 798, 776, 711, 682, 629, 539, 519, 505, 462, 454, 446 cm^{-1}; HRMS (ESI) $C_6H_7O_8P_2$ calcd. 268.9622 [M-H$^+$], found = 268.9622.

(1,2-Phenylenebis(methylene))bis(phosphonate) (**3a**)

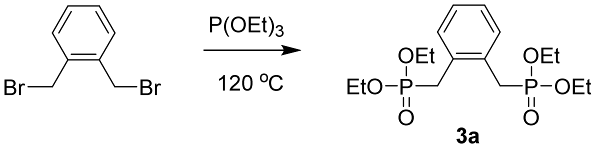

3a

1,2-bis(bromomethyl)benzene (2 g, 7.6 mmol) and triethylphosphite (3.13 ml, 16.7 mmol) were mixed and heated to 120 °C for 3 h. Volatiles were removed under reduced pressure and purified by column chromatography in DCM / MeOH (100:1 → 50:1) to afford **3a** as white solid (2 g, 70%).[10-11] R_f = 0.22 (DCM / MeOH 50:1); Melting point: 46-48 °C; CAS: 42095-05-7; ^{1}H NMR (300 MHz, $CDCl_3$) δ = 1.18 (t, *J*=7.1, 12H), 3.37 (d, *J*=20.3, 4H), 3.82 – 4.04 (m, 8H), 7.14 (qd, *J*=4.3, 2.2, 2H), 7.20 (ddt, *J* = 6.2, 4.1, 1.9, 2H); ^{13}C NMR (75 MHz, $CDCl_3$) δ = 16.29 – 16.85 (m), 31.43 (dd, *J*=137.7, 2.2), 62.25 (t, *J*=3.4), 127.35, 131.19 – 131.28 (m), 131.70; ^{31}P NMR (162 MHz, $CDCl_3$) δ = 27.81 (s, 2P); UV/Vis: λ (nm) = 263, 217; MS (ESI) $C_{16}H_{28}NaO_6P_2$ calcd: 401.1 [M+Na$^+$], found: 401.0

(1,2-Phenylenebis(methylene))bis(phosphonic acid) (**3**)

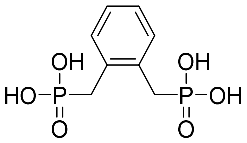

3

3a (0.5 g, 1.32 mmol) was dissolved in 3 M HCl (10 ml) and refluxed at 120 °C for 24 h. After completion of the reaction, volatiles were removed. The obtained solid was washed by refluxing in dichloromethane. After isolation of the product by filtration and removal of the solvent in vacuum, **3** was obtained as white crystals (0.273 g, 75%)[12]. CAS: 42104-58-5; ^{1}H NMR (400 MHz, DMSO-d_6) δ = 3.15 (d, *J*=20.5, 4H), 7.04 – 7.16 (m, 2H), 7.16 – 7.27 (m, 2H), 9.24 (bs, 4H); ^{13}C NMR (101 MHz, DMSO-d_6) δ = 32.61 (d, *J*=130.0), 126.03, 131.12, 132.48 – 132.92 (m); ^{31}P NMR (162 MHz, DMSO-d_6) δ = 22.91 (s); UV/Vis: λ (nm) = 263, 214, 201; IR (KBr): $\tilde{\nu}$ = 3456, 2967, 2936, 2852, 2803, 1630, 1605, 1497, 1454, 1262, 1258, 1215, 1181, 1171, 1128, 1092, 1070, 1052 , 997, 962, 947, 824, 778, 733, 598, 515, 472 , 429, 408 cm^{-1}; HRMS (ESI) $C_8H_{11}O_6P_2$ calcd: 265.0036 [M-H$^+$], found: 265.0039.

N-(3,4-Dihydroxyphenethyl)nonanamide (**4a**)

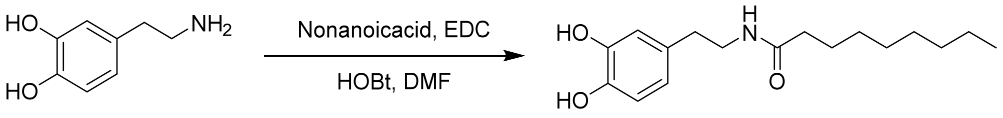

Dopamine **4a**

4a was produced from dopamine (300 mg, 1.96 mmol) and nonanoic acid (250 mg, 1.58 mmol) according to Method 3. After purification by column chromatography in DCM / MeOH (50:1), the product was obtained as a colorless oil (300 mg, 65%). R_f = 0.32 (DCM / MeOH 50:1); ^{1}H NMR (400 MHz, $CDCl_3$) δ = 0.86 (t, J=6.8, 3H), 1.14 – 1.34 (m, 10H), 1.56 (p, J=7.3, 2H), 2.05 – 2.20 (m, 2H), 2.66 (t, J=7.0, 2H), 3.37 – 3.53 (m, 2H), 5.85 (t, J=5.9, 1H), 6.53 (dd, *J* = 8.0, 2.1, 1H), 6.73 (d, J=2.1, 1H), 6.80 (d, J=8.0, 1H); ^{13}C NMR (101 MHz, $CDCl_3$) δ = 14.19, 22.74, 25.87, 29.22, 29.32, 29.35, 34.99, 36.91, 41.07, 115.32, 115.58, 120.45, 130.57, 143.35, 144.65, 174.56; UV/Vis: λ (nm) = 283, 204; IR (Film): $\tilde{\nu}$ = 3480, 3339, 3186, 2950, 2925, 2854, 2726, 2643, 2413, 1842, 1660, 1602, 1555, 1530, 1455, 1443, 1384, 1281, 1254, 1222, 1193, 1150, 1114, 1062, 1048, 1027, 959, 940, 930, 922, 856, 814, 786, 757, 722, 664, 636, 616, 588, 560, 530, 489, 461, 452, 432, 407 cm^{-1}; MS (ESI) $C_{17}H_{27}NO_3$ calcd: 292.2 [M-H^+], found: 292.1

Tetrabenzyl (4-(2-nonanamidoethyl)-1,2-phenylene) bis(phosphate) (**4b**)

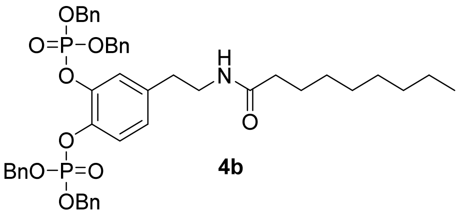

4b

4b was synthesized from **4a** (150 mg, 0.51 mmol) according to Method 1. After purification by column chromatography in DCM/MeOH (100:1 → 50:1), the product was obtained as colorless oil (300 mg, 80%). R_f = 0.37 (DCM/MeOH 50:1), ^{1}H NMR (400 MHz, $CDCl_3$) δ = 0.87 (t, *J*=6.7, 3H), 1.26 (d, *J*=4.5, 9H), 1.54 – 1.63 (m, 2H), 2.06 – 2.19 (m, 3H), 2.69 (t, *J*=6.8, 2H), 3.40 (q, *J*=6.6, 2H), 5.09 (dd, *J*=8.1, 1.8, 8H), 5.44 (t, *J*=5.8, 1H), 6.89 (dd, *J*=8.0, 1.8, 1H), 7.12 (s, 1H), 7.26 (m, 21H); ^{13}C NMR (101 MHz, $CDCl_3$) δ = 14.22, 22.76, 25.85, 29.28, 29.45, 31.95, 35.17, 36.87, 40.39, 70.23 (dd, *J*=5.9, 3.0), 121.75 (d, *J*=2.3), 121.96 (d, *J*=2.3), 126.07, 128.09, 128.11, 128.67, 128.69, 128.72, 128.75, 135.46 (d, *J*=7.1), 137.31, 140.02 (t, *J*=6.6), 141.43 (t, *J*=6.5), 173.30; ^{31}P NMR (162 MHz, $CDCl_3$) δ = -5.06 (s, 1P), -5.26 (s, 1P); MS (ESI) $C_{45}H_{53}NNaO_9P_2$ calcd: 836.3 [M+Na^+], found: 836.3.

4-(2-Nonanamidoethyl)-1,2-phenylene bis(dihydrogen phosphate) (**4**)

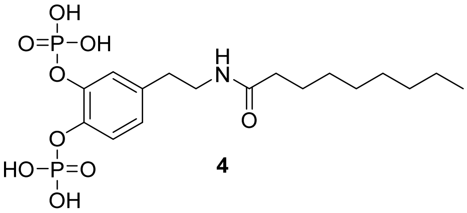

4

4 was obtained from **4b** (250 mg, 0.31mmol) according to Method 2. Lyophilization yielded the product as an oil (140 mg, 99%); ^{1}H NMR (300 MHz, DMSO-d_6) δ = 0.84 (t, 3H), 1.11 – 1.32 (m, 10H), 1.36 – 1.57 (m, 2H), 2.01 (t, *J*=7.4, 2H), 2.61 (t, *J*=7.4, 2H), 3.20 (q, *J*=6.4, 2H), 5.60 – 6.20 (bs, P(O)OH + H_2O), 6.92 (dd, 1H), 7.16 (s, 1H), 7.22 (d, *J*=8.3, 1H), 7.85 (t, *J*=5.5, 1H); ^{13}C NMR (75 MHz, DMSO-d_6) δ = 13.99, 22.10, 25.28, 28.61, 28.71, 28.77, 31.29, 34.62, 35.43, 121.87, 122.22, 124.52, 135.98, 140.88 (t, *J*=6.1), 142.34 (t, *J*=5.9), 160.65, 172.16; ^{31}P NMR (162 MHz, DMSO-d_6) δ = -4.58 (s, 1P), -4.71 (s, 1P); UV/Vis: λ (nm) = 268, 211; IR

(Film): $\tilde{\nu}$ = 3412, 2923, 2853, 1692, 1659, 1651, 1643, 1632, 1614, 1555, 1513, 1505, 1384, 1069, 1034, 724, 597, 507, 497, 489, 467, 459, 445 cm^{-1}; HRMS (ESI) $C_{17}H_{28}NO_9P_2$ calcd: 452.1245 [M-H$^+$], found: 452.1246.

(3,4-dihydroxyphenyl)-N,N-dioctylpropanamide 3-(3,4-dihydroxyphenyl) propanoate (**5a**)

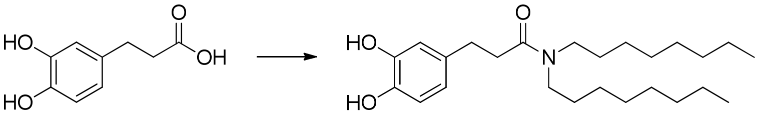

5a

3,4-Dihydroxyhydrocinnamic acid (hydrocaffeic acid) (300 mg, 1.7 mmol) was dissolved in anhydrous DMF (6 mL). The reaction was cooled down to 0 °C and 1-hydroxybenzotriazole hydrate (HOBt, 223 mg, 1.7 mmol) and *N*,*N'*-di-cyclohexylcarbodiimide (DCC, 1.7 mmol, 340 mg) were added to a stirred solution. After 1 h, dioctylamine (0.5 mL, 1.7 mmol) was added at 0 °C. After overnight reaction at room temperature, 10 mL of water was added. The reaction mixture was then extracted three times with 15 mL diethyl ether. The combined organic layers were washed with saturated $NaHCO_3$ solution, dried over Na_2SO_4 and concentrated *in vacuo*. The product was purified by column chromatography in DCM/MeOH (100:1 → 20:1) to afford **5a (**380 mg, 57 %); R_f = 0.33 (DCM/MeOH 20:1); ^{1}H NMR (400 MHz, $CDCl_3$): δ = 0.81 – 0.93 (bs, 6H), 1.26 (bs, 20H), 1.40 – 1.59 (m, 4H), 2.63 (t, *J*=7.6, 2H), 2.86 (t, *J*=7.6, 2H), 3.14 (t, *J*=8.0, 2H), 3.29 (t, *J*=7.8, 2H), 6.56 (dd, *J*=8.1, 2.0, 1H), 6.72 – 6.86 (m, 2H); ^{13}C NMR (101 MHz, $CDCl_3$): δ = 14.2, 22.7, 22.8, 27.0, 27.2, 27.8, 29.1, 29.3, 29.4, 29.5, 31.5, 31.9, 35.2, 46.9, 48.7, 115.7, 119.9, 132.7, 143.2, 144.4, 173.3; UV/Vis: λ (nm) = 284, 212; IR (KBr): $\tilde{\nu}$ = 3535, 3360, 2954, 2926, 2855, 2359, 1761, 1739, 1601, 1518, 1487, 1466, 1447, 1375, 1281, 1259, 1195, 1149, 1111, 1012, 954, 866, 810, 786, 721, 636, 590 cm^{-1}; MS (ESI) $C_{25}H_{43}NO_3$ calcd: 406.3 [M+H$^+$], found: 406.5.

Dibenzyl 4-(3-(dioctylamino)-3-oxopropyl)-1,2-phenylene diphosphate (**5b**)

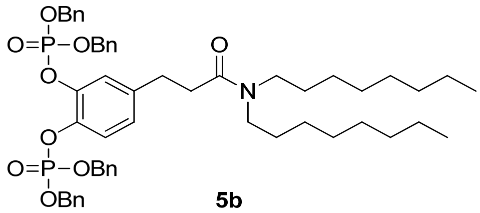

5b

5b was produced from **5a** (90 mg, 0.22 mmol) according to Method 1. The product was purified by column chromatography in hexane / ethyl acetate (5:1 → 2:5) yielding (170 mg, 83 %) as colorless oil. R_f = 0.38 (hexane/ethyl acetate 2:5); ^{1}H NMR (400 MHz, $CDCl_3$) δ = 0.87 (t, *J*=6.7, 6H), 1.26 (bs, 20H), 1.49 (bs, 4H), 2.52 – 2.60 (m, 2H), 2.86 – 2.95 (m, 2H), 3.02 – 3.22 (m, 2H), 3.22 – 3.41 (m, 2H), 5.07 – 5.12 (m, 8H), 6.96 (dd, *J*=8.5, 1.0, 1H), 7.15 – 7.19 (m, 1H), 7.22 – 7.30 (m, 21H). ^{13}C NMR (101 MHz, cdcl$_3$) δ = 14.21, 22.75, 29.35, 29.47, 31.05, 31.90, 34.68, 70.19 (dd, *J*=5.9, 4.3), 121.65, 125.86, 128.08, 128.10, 128.66, 128.70, 135.51 (d, *J*=7.2), 139.61, 139.75 (t, *J*=6.5), 141.29 (t, *J*=6.5), 171.59; ^{31}P NMR (162 MHz, $CDCl_3$) δ = -5.02 (s, 1P), -5.31 (s, 1P); UV/Vis: λ [nm] = 264, 226; IR (KBr): $\tilde{\nu}$ = 3065, 3034, 2954, 2926, 2855, 1736, 1642, 1592, 1510, 1456, 1425, 1380, 1288, 1214, 1154, 1122, 1081, 1036, 1015,

1000, 952, 886, 823, 740, 697, 639, 601, 500, 457 cm^{-1}; MS (ESI) $C_{53}H_{69}NNaO_9P_2$ calcd: 948.4 [M+Na$^+$], found: 948.4.

4-(3-(Dioctylamino)-3-oxopropyl)-1,2-phenylene bis(dihydrogen phosphate) (**5**)

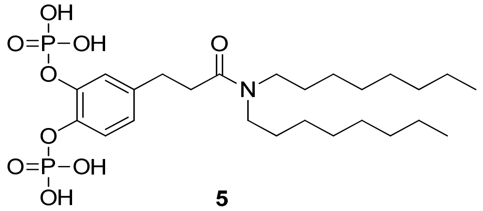

5

5 was obtained from **5b** (130 mg, 0.14 mmol) according to Method 2. After lyophilization, the product was obtained as a hygroscopic white solid (80 mg, 99%); ^{1}H NMR (400 MHz, DMSO-*d*$_6$) δ = 0.78 – 0.94 (m, 6H), 1.02 – 1.52 (m, 24H), 2.51 – 2.57 (m, 2H), 2.73 (t, *J*=7.7, 2H), 3.05 – 3.35 (m, 4H), 5.45 (bs, OH), 6.92 (d, *J*=8.0, 1H), 7.00 – 7.28 (m, 2H); ^{13}C NMR (75 MHz, DMSO-*d*$_6$) δ = 13.98, 22.09, 26.23, 27.34, 28.64, 30.36, 31.26, 33.93, 45.14, 47.10, 104.32, 122.59, 123.97, 137.69, 170.44; ^{31}P NMR (162 MHz, DMSO-*d*$_6$) δ = -4.34 (s, 1P), -4.57 (s, 1P); UV/Vis: λ (nm) = 275, 269, 202; IR (KBr): $\tilde{\nu}$ = 3628, 3434, 2955, 2927, 2856, 2731, 2360, 2344, 2324, 1732, 1718, 1699, 1684, 1636, 1615, 1589, 1559, 1541, 1509, 1467, 1427, 1375, 1280, 1214, 1155, 1120, 984, 822, 723, 502 cm^{-1}; HRMS (ESI) $C_{25}H_{44}NO_9P_2$ calcd: 564.2497 [M-H$^+$], found: 564.2499.

(2E,4E)-5-(3,4-Dihydroxyphenyl)-1-(piperidin-1-yl)penta-2,4-dien-1-one (**6a**)

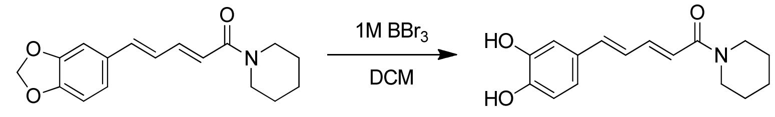

Piperine **6a**

Piperine (500 mg, 1.75 mmol) was dissolved in 4 mL of anhydrous DCM, and a 1M solution of BBr_3 in DCM (2.2 mL) was added at 0 °C. After stirring for 16 h at room temperature, the solvent was removed. The product was purified by column chromatography in DCM/MeOH (200:1), to afford **6a** as light yellow powder (238 mg, 50 %); R_f = 0.2 (DCM/MeOH 19:1); ^{1}H NMR (400 MHz, CD_3OD): δ =1.54 – 1.64 (m, 4H), 1.66 – 1.74 (m, 2H), 3.56 – 3.69 (m, 4H), 6.57 (d, *J* = 14.6 Hz, 1H), 6.66 – 6.91 (m, 4H), 6.96 (d, *J* = 2.1 Hz, 1H), 7.32 (dd, *J* = 14.6, 9.8 Hz, 1H); ^{13}C NMR (100 MHz, CD_3OD): δ = 25.5, 26.9, 27.9, 44.5, 114.4, 116.5, 119.6, 121.2, 125.2, 130.0, 141.0, 145.0, 146.6, 147.8, 167.9; UV/Vis: λ (nm) = 353, 345, 204; MS (ESI) $C_{16}H_{19}NNaO_3$ calcd: 296.1 [M+Na$^+$], found: 296.1.

Dibenzyl 4-((1*E*,3*E*)-5-oxo-5-(piperidin-1-yl)penta-1,3-dienyl)-1,2-phenylene diphosphate (**6b**)

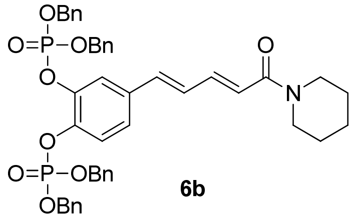

6b

6a (150 mg, 0.55 mmol) was converted into **6b** according to Method 1. The crude product was purified by column chromatography in DCM/methanol (200:1 → 100:1), to yield **6b** (221 mg, 51 %) as a colorless oil. R_f = 0.33 (DCM/methanol 19:1); ^{1}H NMR (300 MHz, $CDCl_3$): δ =1.40 – 1.83 (m, 6H), 3.59 (m, 4H), 5.04 – 5.13 (m, 8H), 6.47 (d, J = 14.7 Hz, 1H), 6.59 – 6.79 (m, 2H), 7.10 (dd, J = 8.4, 1.7 Hz, 1H), 7.20 – 7.46 (m, 23H); ^{13}C NMR (75 MHz, $CDCl_3$): δ =24.78, 26.93, 43.40, 70.32 (d, *J*=5.9), 104.88, 119.36, 121.96, 124.60, 128.11, 128.14, 128.75, 134.70, 136.18, 141.71, 165.25, 204.36; ^{31}P NMR (162 MHz, $CDCl_3$): δ= -5.32, -5.34; UV/Vis: λ (nm) = 316, 234; MS (ESI) $C_{44}H_{45}NNaO_9P_2$ calcd: 816.3 [M+Na$^+$], found: 816.3.

4-(5-Oxo-5-(piperidin-1-yl)pentyl)-1,2-phenylene bis(dihydrogen phosphate) (**6**)

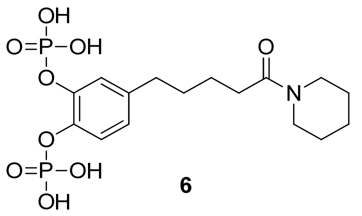

6

6 was produced from **6b** (250 mg, 0.3 mmol) according to Method 2. The product was obtained as a light yellow solid (140 mg, 99%); Melting point: 137-150 °C; ^{1}H NMR (400 MHz, D_2O) δ = 1.40 – 1.72 (m, 10H), 2.39 (s, 2H), 2.58 (s, 2H), 3.41 (s, 4H), 6.99 (d, *J*=7.6, 1H), 7.17 (s, 1H), 7.22 (d, *J*=8.1, 1H); ^{13}C NMR (101 MHz, D_2O) δ = 23.73, 24.66, 29.97, 32.73, 34.04, 121.76, 121.96, 125.24, 140.09, 140.63 (t, *J*=6.7), 142.49 (t, *J*=6.1), 174.47; ^{31}P NMR (162 MHz, D_2O) δ = -3.20 (s, 1P), -3.05 (s, 1P); UV/Vis: λ (nm) = 269, 203; IR (KBr): $\tilde{\nu}$ = 3650, 3436, 2940, 2859, 2360, 2338, 2325, 1711, 1610, 1589, 1508, 1446, 1422, 1281, 1211, 1151, 1119, 1012, 980, 851, 822, 722, 638, 512, 503, 462 cm^{-1}; HRMS (ESI) $C_{16}H_{24}NO_9P_2$ calcd: 436.0932 [M-H$^+$], found: 436.0933.

(9H-Fluoren-9-yl) methyl (3,4-dihydroxyphenethyl)carbamate (**7a**)

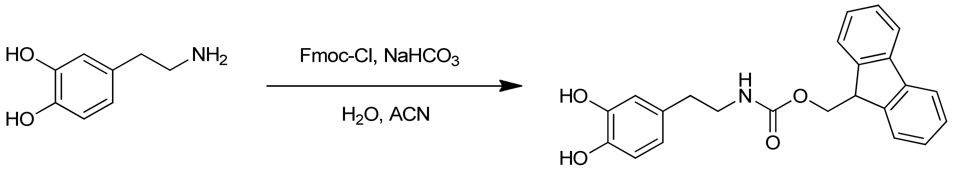

Dopamine **7a**

7a was synthesized from dopamine (1 g, 5.2 mmol) according to method 4. The crude material was purified by column chromatography (hexane/ethyl acetate 2:1) to obtain pure product **7a** (1.5 g, 77%)[13]; CAS: 248596-35-2. R_f = 0.27 (hexane/ethyl acetate 2:1); ^{1}H NMR (400 MHz, DMSO-*d*$_6$) δ = 2.50 – 2.56 (m, 2H), 3.11 (q, *J*=6.4, 2H), 4.20 (d, *J*=6.6, 1H), 4.27 (d, *J*=7.0, 2H), 6.41 (dd, *J*=8.0, 1.6, 1H), 6.62 (d, *J*=8.0, 1H), 7.31 (td, *J*=7.4, 1.1, 3H), 7.40 (t, *J*=7.4, 2H), 7.66 (d, *J*=7.4, 2H), 7.87 (d, *J*=7.5, 2H), 8.63 (s, 1H), 8.73 (s, 1H); ^{13}C NMR (101 MHz, DMSO-*d*$_6$) δ = 35.31, 38.87, 42.31, 109.70, 115.51, 116.03, 119.13, 119.99, 121.35, 127.26, 128.89, 130.28, 137.40, 139.39, 142.55, 143.50, 145.08, 157.44.

(9H-Fluoren-9-yl)methyl (3,4-bis((bis(benzyloxy)phosphoryl)oxy)phenethyl) carbamate (**7b**)

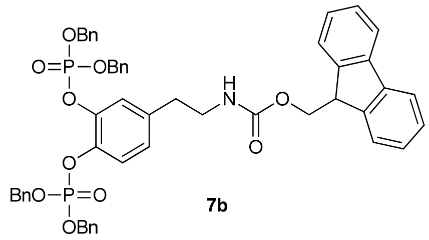

7b

7b was synthesized from **7a** (500 mg, 1.33 mmol) according to Method 1 and purified by column chromatography in hexane/ethyl acetate (3:1 → 1:1) as colorless oil (1.05 g, 88% yield); R_f = 0.18 (hexane/ethyl acetate 1:1); ^{1}H NMR (400 MHz, $CDCl_3$) δ = 2.70 (t, *J*=6.4, 2H), 3.34 (d, *J*=6.4, 2H), 4.22 (t, *J*=6.6, 1H), 4.42 (d, *J*=6.7, 2H), 4.76 (s, 1H), 5.11 (dd, *J*=8.1, 2.4, 9H), 6.87 (d, *J*=8.1, 1H), 7.13 (s, 1H), 7.22 – 7.46 (m, 27H), 7.59 (d, *J*=7.4, 2H), 7.77 (d, *J*=7.5, 2H); ^{13}C NMR (101 MHz, $CDCl_3$) δ= 35.52, 42.08, 47.40, 66.69, 70.22 (dd, *J*=2.9), 120.09, 121.71, 121.92, 125.16, 126.14, 127.18, 127.77, 128.10, 128.12, 128.22, 128.67, 128.70, 128.72, 135.45, 135.51, 136.99, 140.08, 141.44, 144.05, 156.35; ^{31}P NMR (162 MHz, $CDCl_3$) δ = -5.29 (s, 1P), -5.06 (s, 1P); UV/Vis: λ (nm) = 300, 289, 265, 212; IR (Film): $\tilde{\nu}$ = 3423, 3320, 3089, 3064, 3034, 2951, 2895, 1720, 1607, 1591, 1509, 1477, 1454, 1425, 1382, 1281, 1251, 1214, 1151, 11235, 1106, 1080, 1015, 1000, 963, 8/89, 823, 741, 697, 640, 620, 600, 507, 499, 427 cm^{-1}; MS (ESI) $C_{51}H_{47}NNaO_{10}P_2$ calcd: 918.3 [M+Na$^+$], found: 918.2.

(9H-Fluoren-9-yl)methyl (3,4-bis(phosphonooxy)phenethyl)carbamate tetrasodium salt (**7**)

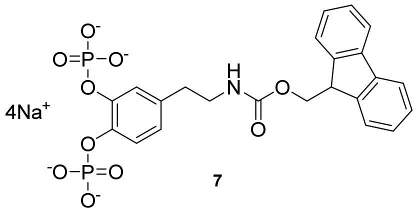

7

7b (250 mg, 0.28 mmol) was treated according to method 2. The obtained phosphoric acid (75 mg, 0.14 mmol) was dissolved in 3 ml H_2O and mixed with an aqueous solution of $NaHCO_3$ (47 mg, 0.56 mmol). The clear solution was filtered through cotton (4 – 6 cm in a glass pipette). The cotton was washed with 3-5 ml of H_2O and lyophilized again to afford **7** as tetra sodium salt (77 mg, 88 %); Decomposition point: >250 °C charred without melting; ^{1}H NMR (400 MHz, D_2O) δ = 2.47 (s, 2H), 3.05 – 3.22 (m, 2H), 4.18 (s, 1H), 4.42 (d, *J*=4.6, 2H), 6.55 (d, *J*=7.5, 1H), 7.10 – 7.20 (m, 2H), 7.24 – 7.47 (m, 4H), 7.57 (d, *J*=7.4, 2H), 7.81 (d, *J*=7.5, 3H); ^{13}C NMR (75 MHz, D_2O) δ = 34.83, 40.75, 47.26, 66.16, 120.31, 120.48, 120.70, 122.46, 125.18, 127.61, 128.15, 130.30, 133.13, 141.10, 143.08 (t, *J*=6.1), 144.59 (t, *J*=6.1), 144.01, 158.42; ^{31}P NMR (162 MHz, D_2O) δ = 1.48 (s, 1P), 1.57 (s, 1P); UV/Vis: λ (nm) = 299, 265, 214; IR (KBr): $\tilde{\nu}$ = 3751, 3735, 3404, 2212, 2146, 1696, 1583, 1508, 1478, 1449, 1422, 1338, 1278, 1221, 1122, 991, 967, 931, 879, 833, 818, 783, 760, 741, 703, 620, 593, 585, 567, 556, 504, 527, 499, 492, 477, 462, 443, 427, 413 cm^{-1}; HRMS (ESI) $C_{23}H_{22}NO_{10}P_2$ calcd: 534.0724 [M-H$^+$], found: 534.0717.

Methyl (*S*)-2-((((9H-fluoren-9-yl)methoxy)carbonyl)amino)-3-(3,4-dihydroxyphenyl)propanoate (**8a**)

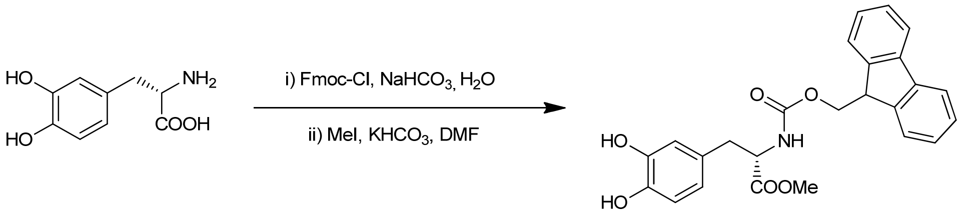

L-DOPA **8a**

Fmoc-L-Dopa was obtained from commercially available L-Dopa (200mg, 1.0 mmol) according to method 4. This product was used without further purification. To the solution of Fmoc-L-Dopa (400 mg, 0.95 mmol) in 5 ml of dry DMF were added $KHCO_3$ (142 mg, 1.42 mmol) and methyl iodide (70 µl, 1.1 mmol), and the reaction was stirred for 4 h at room temperature. The crude product was purified by column chromatography in hexane / ethyl acetate (4:1 → 2:1) to obtain **8a** as light brown oil (272 mg, 62% over 2 steps). ^{1}H NMR (300 MHz, DMSO-d_6) δ = 2.72 – 2.86 (m, 2H), 3.59 (s, 3H), 3.90 – 4.44 (m, 3H), 6.47 (dd, *J*=8.1, 2.1, 1H), 6.53 – 6.72 (m, 2H), 7.22 – 7.35 (m, 2H), 7.35 – 7.44 (m, 2H), 7.64 (t, *J*=7.4, 2H), 7.79 (d, *J*=8.1, 1H) 7.86 (d, *J*=7.5, 2H), 7.93 (s, 1H), 8.73 (s, 2H); ^{13}C NMR (75 MHz, DMSO-d_6) δ = 36.04, 46.58, 51.85, 55.95, 65.68, 115.36, 116.38, 119.80, 120.10, 125.23, 127.08, 127.63, 128.12, 140.69, 143.73, 143.91, 144.96, 155.89, 172.53; MS (ESI) $C_{25}H_{23}NNaO_6$ calcd: 456.4 [M+Na$^+$], found: 456.3.

Methyl (*S*)-2-((((9H-fluoren-9-yl)methoxy)carbonyl)amino)-3-(3,4-bis((bis(benzyloxy)-phosphoryl) oxy)phenyl)propanoate (**8b**)

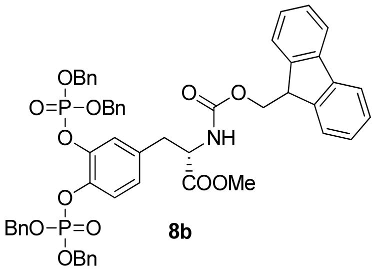

8b

8b was produced from **8a** (120 mg, 0.28 mmol) according to Method 1. Purification of the crude product by column chromatography in hexane/ethyl acetate (1:1) gave the product as a colorless oil (200 mg, 75%); R_f = 0.37 (hexane/ethyl acetate 1:1); ^{1}H NMR (400 MHz, DMSO-d_6) δ = 2.80 – 3.08 (m, 2H), 3.62 (s, 3H), 4.10 – 4.30 (m, 4H), 4.96 – 5.23 (m, 8H), 7.11 (d, *J*=7.8, 1H), 7.18 – 7.43 (m, 26H), 7.62 (t, *J*=7.5, 2H), 7.86 (d, *J*=7.5, 2H), 7.93 (d, *J*=8.2, 1H); ^{13}C NMR (101 MHz, DMSO-d_6) δ = 35.56, 46.54, 51.99, 55.26, 65.67, 69.53 (t, *J*=5.5), 120.07, 121.02, 122.17, 125.14 (d, *J*=2.8), 126.67, 127.02, 127.59, 127.86, 127.93, 128.01, 128.37, 128.44, 128.48, 128.84, 129.45, 135.41 (dd, *J*=7.0, 3.2), 135.66, 139.68 (t, *J*=6.2), 140.52 (t, *J*=6.5), 143.62, 143.70, 155.91, 172.00; ^{31}P NMR (162 MHz, DMSO-d_6) δ = -5.27 (s, 1P), -5.36 (s, 1P); UV/Vis: λ (nm) = 300, 289, 265, 217; IR (Film): $\tilde{\nu}$ = 3481, 3421, 3298, 3065, 3034, 2953, 2897, 2360, 1722, 1672, 1654, 1637, 1594, 1540, 1509, 1455, 1428, 1384, 1280, 12147, 1180, 1158, 1124, 1081, 1035, 1018, 1000, 964, 881, 787, 759, 741, 697, 598, 510 ,490, 470, 458 cm^{-1}; MS (ESI) $C_{53}H_{49}NNaO_{12}P_2$ calcd: 976.3 [M+Na$^+$], found: 976.4.

Synthesis of methyl (*S*)-2-((((9H-fluoren-9-yl)methoxy)carbonyl)amino)-3-(3,4-bis(phosphonooxy)phenyl)propanoate (**8**)

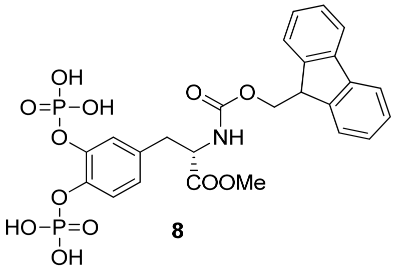

8

8 was produced from **8b** (120 mg, 0.13 mmol) according to Method 2 to afford a pale yellow hygroscopic solid (60 mg, 81 %); ^{1}H NMR (400 MHz, DMSO-d_6) δ = 2.83 – 3.01 (m, 2H), 3.61 (s, 3H), 4.19 – 4.24 (m, 4H), 4.3 – 5.0 (bs, 4P-OH + H_2O), 6.94 (dd, *J* = 8.1 Hz, 2.1 Hz, 1H), 7.13 – 7.21 (m, 2H), 7.30 (q, *J*=7.2, 2H), 7.39 (t, *J*=7.2, 1H), 7.65 (t, *J*=7.6, 2H), 7.86 (d, *J*=7.6, 2H), 8.40 (s, 1H); ^{31}P NMR (162 MHz, DMSO-d_6) δ = 0.14, 0.25; IR (KBr): $\tilde{\nu}$ = 3434, 2955, 2926, 2854, 2360, 2338, 1705, 1671, 1634, 1509, 1449, 1427, 1385, 1338, 1279, 1252, 1217, 1158, 1123, 1081, 953, 906, 760, 741, 699, 621, 572, 534, 512 cm^{-1}; HRMS (ESI) $C_{25}H_{24}NO_{12}P_2$ calcd: 592.0779 [M-H$^+$], found: 592.0779.

Synthesis of (9*H*-Fluoren-9-yl) methyl 3,4-dihydroxybenzylcarbamate (**9a**)

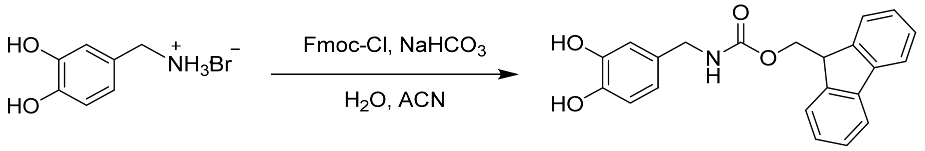

9a

(4-(Aminomethyl)benzene-1,2-diol hydrobromide (200 mg, 0.9 mmol) was dissolved in 2 mL of water, and 2 ml of saturated $NaHCO_3$ was added at 0 °C. After 15 min, 9-fluorenylmethoxycarbonyl chloride (Fmoc-Cl) (282 mg, 1.1 mmol, in 3 ml CH_3CN) was added and a white precipitate was formed. The reaction mixture was stirred at 0 °C for 1.5 h and then stirred at room temperature for an additional 16 h. After completion of the reaction, 10 mL of water and 5 mL of 1M HCl were added. The mixture was extracted with ethyl acetate (3 x 20 mL). The combined organic phases were dried over Na_2SO_4 and concentrated *in vacuo*. The product was purified by column chromatography in DCM/methanol (50:1) to afford **9a** as oil (160 mg, 49 %); R_f = 0.24 (DCM/MeOH 50:1); ^{1}H NMR (400 MHz, $CDCl_3$ + CD_3OD) δ = 4.16 (m, 3H), 4.36 (d, *J* = 4, 2H), 6.59 (d, *J* = 12, 1H,), 6.72 (m, *J*=8.0, 2H), 7.26 (t, *J* = 4, 2H), 7.35 (t, *J* = 4, 2H), 7.55 (d, *J* = 8, 2H), 7.71 (d, *J* = 8, 2H); ^{13}C NMR (100 MHz, $CDCl_3$ + CD_3OD) δ = 44.7, 47.4, 66.9, 114.9, 115.3, 119.5, 120.1, 125.2, 127.2, 127.8, 130.5, 141.4, 144.1, 157.2; UV/Vis: λ (nm) = 300, 288, 265, 211; IR (KBr): $\tilde{\nu}$ = 3424, 2925, 2953, 1694, 1646, 1635, 1523, 1449, 1384, 1264, 1129, 1114, 759, 741, 621, 589, 570, 538, 526, 513, 503 cm^{-1}; MS (ESI) $C_{50}H_{45}NO_{10}P_2$ calcd: 360.1 [M-H$^+$], found: 360.1.

(9*H*-Fluoren-9-yl) methyl 3, 4- bis (bis(benzyloxy)phosphoryloxy) benzyl-carbamate (**9b**)

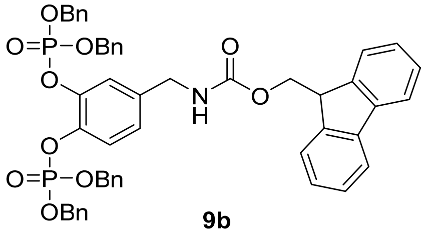

9b

9b was synthesized from **9a** (140 mg, 0.4 mmol) according to Method 1. After purification by column chromatography (hexane/ethyl acetate 3:1 → 1:1), the product was obtained in as a colorless oil (308 mg, 90%); R_f = 0.34 (hexane/ethyl acetate 1:1); ^{1}H NMR (400 MHz, $CDCl_3$): δ = 4.22 (m, 2H), 4.46 (d, *J* = 6.7, 2H), 4.98 (t, *J* = 6.2, 1H), 5.10 (dd, *J*=8.1, 2.2, 8H), 6.98 (d, *J* = 8.3, 1H), 7.17 (s, 1H), 7.20 – 7.36 (m, 23H), 7.40 (t, *J* = 7.4, 2H), 7.59 (d, *J* = 7.4, 2H), 7.76 (d, *J* = 7.5, 2H); ^{13}C NMR (101 MHz, $cdcl_3$) δ = 44.30, 47.40, 66.86, 70.28 (dd, *J* = 5.8, 3.4),120.11, 120.88 (d, *J*=2.3), 121.94 (d, *J*=1.9), 125.12, 127.20, 127.83, 128.11, 128.15, 128.67, 128.73, 135.45 (d, *J*=7.1), 136.49, 140.74 (t, *J*=6.5), 141.46, 143.98, 156.40; ^{31}P NMR (162 MHz, $CDCl_3$): δ = -5.30 (s, 1P), -5.15 (s, 1P); IR (KBr): $\tilde{\nu}$ = 3419, 3313, 3089, 3065, 3034, 2953, 2895, 2360, 2342, 2323, 1721, 1654, 1607, 1594, 1538, 1510, 1477, 1455, 1428, 1383, 1357, 1281, 1214, 1158, 1122, 1081, 1017, 1000, 884, 822, 759, 741, 697, 620, 602, 511, 500, 492, 459, 426 cm^{-1}; MS (ESI) $C_{50}H_{45}NO_{10}P_2$ calcd: 904.2 [M+Na$^+$], found: 904.3.

(9H-Fluoren-9-yl) methyl (3,4-bis(phosphonooxy)benzyl)carbamate (**9**)

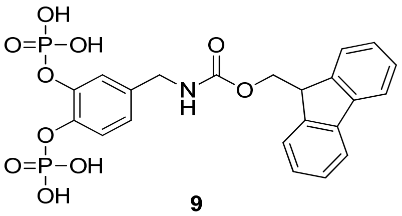

9

9 was produced from **9b** (170 mg, 0.19 mmol) according to Method 2 as pale yellow powder (100 mg, 75%); Melting point: 132-133 °C; ^{1}H NMR (400 MHz, DMSO-d_6) δ = 4.12 (d, *J*=6.1, 2H), 4.25 (t, *J*=7.0, 1H), 4.33 (d, *J*=7.0, 2H), 6.92 (d, *J*=8.5, 1H), 7.20 (s, 2H), 7.35 (q, *J*=8.0, 7.5, 2H), 7.42 (q, *J*=5.9, 4.3, 2H), 7.71 (d, *J*=7.5, 2H), 7.90 (d, *J*=7.5, 3H), 8.16 (s, 1H); ^{13}C NMR (101 MHz, DMSO-d_6) δ = 43.21, 46.73, 65.44, 120.07, 121.41, 122.39, 122.75, 125.19, 127.07, 127.58, 135.90, 140.71, 143.87, 156.28; ^{31}P NMR (162 MHz, CD_3OD) δ = -3.15 (s); UV/Vis: λ (nm) = 300, 289, 265, 210; IR (KBr): $\tilde{\nu}$ = 3423, 3067, 3019, 2958, 2926, 2892, 1693, 1646, 1595, 1543, 1510, 1449, 1429, 1284, 1272, 1214, 1161, 1120, 976, 822, 759, 740, 583, 521, 507, 499, 466, 427 cm^{-1}; HRMS (ESI) $C_{22}H_{20}NO_{10}P_2$ calcd: 520.0568 [M-H$^+$], found: 520.0563.

Methyl 7-(5,5-difluoro-1,3,7,9-tetramethyl-5H-4λ⁴,5λ⁴-dipyrrolo[1,2-c:2',1'-f][1,3,2]diazaborinin-10-yl)heptanoate (**10a**)

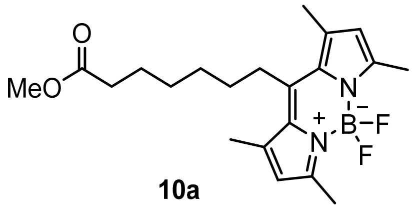

10a

Synthesis of **10a** was carried out in a variation of the published procedure.[14] To a solution of 2,4-dimethyl-1*H*-pyrrole (1.84 g, 19.3 mmol) in dry DCM (40 mL), methyl 8-chloro-8-oxooctanoate (2 g, 9.67 mmol) was added dropwise over a period of 10 min at room temperature. The deep red solution was refluxed for 4 h and poured into hexane (50 mL). After cooling, the solvent was evaporated in vacuo. The solid was dissolved in dry DCM (20 mL), triethylamine was added (2.7 mL, 19.3 mmol), and the solution was stirred for 15 min at room temperature. Subsequently, BF_3-Et_2O (5.48 mL, 38.6 mmol) was added dropwise, and the mixture was stirred for 2 h at room temperature. The deep red solution was poured into sat. $NaHCO_3$ solution. The organic phase was separated, washed with saturated aqueous Na_2CO_3 solution, and dried over Na_2SO_4. After removal of the solvent under reduced pressure and column chromatography in hexane/DCM (4:1 → 1:1 → 2:3), **10a** is obtained as an orange-green fluorescing solid (2 g, 50%).[14-15] Melting point: 128-130 °C; R_f = 0.33 (hexane/DCM 2:3); ^{1}H NMR (300 MHz, $CDCl_3$) δ = 1.32 – 1.56 (m, 4H), 1.64 (tt, *J*=14.3, 7.1, 4H), 2.31 (t, *J*=7.4, 2H), 2.40 (s, 6H), 2.51 (s, 6H), 2.84 – 3.01 (m, 2H), 3.66 (s, 3H), 6.04 (s, 2H); ^{13}C NMR (75 MHz, $CDCl_3$) δ = 14.57, 16.51, 24.99, 28.50, 29.08, 30.17, 31.86, 34.06, 51.64, 121.72, 131.54, 140.38, 146.51, 153.91, 165.97, 174.14; ^{19}F NMR (282 MHz, $CDCl_3$) δ = -147.10 (dd, *J*=65.2, 31.6); UV/Vis: λ (nm) = 495, 358, 304, 241; IR (KBr): $\tilde{\nu}$ = 3448, 2931, 2867, 2857, 1735, 1640, 1552, 1535, 1510, 1474, 1464, 1447, 1433, 1408, 1370, 1324, 1307, 1276, 1250, 1225, 1205, 1162, 1112, 1082, 1060, 1023, 988, 838, 810, 730, 716, 581, 514, 482 cm^{-1}; MS (ESI) $C_{21}H_{29}BF_2N_2NaO_2$ calcd: 413.2 [M+Na$^+$], found: 413.2.

7-(5,5-Difluoro-1,3,7,9-tetramethyl-5H-4λ⁴,5λ⁴-dipyrrolo[1,2-c:2',1'-f][1,3,2]diazaborinin-10-yl)heptanoic acid (**10b**)

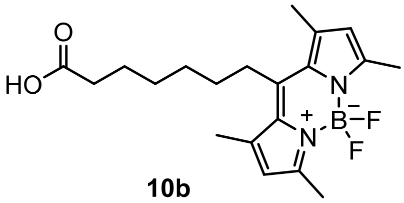

10b

Synthesis of **10b** was carried out essentially as described.[14] To the solution of **10a** (300 mg, 0.77 mmol) in isopropanol (10 mL), an aqueous solution of 0.1 M KOH was added dropwise at 0 ºC, and the mixture was stirred for 2 h at room temperature. The solution was diluted with 100 mL of water and acidified with 1 N HCl to pH = 1.5. The solution was extracted with ethyl acetate (3 x 30 mL), and the combined organic phases were dried over Na_2SO_4. After removal of the solvent under reduced pressure and column chromatography (hexane / ethyl acetate 3:2), **10b** was obtained as an orange solid (287 mg, 99%)[14]; Melting point: 198-200 °C; R_f = 0.28 (hexane/ethyl acetate 3:2); ^{1}H NMR (400 MHz, $CDCl_3$) δ = 1.38 – 1.56 (m, 4H), 1.59 – 1.72 (m, 4H), 2.36 (t, *J*=7.4, 2H), 2.40 (s, 6H), 2.51 (s, 6H), 2.83 – 3.02 (m, 2H), 6.05 (s, 2H); ^{13}C NMR (75 MHz, $CDCl_3$) δ = 14.58, 16.53, 24.72, 28.48, 28.98, 30.13, 31.83, 33.94, 121.75, 131.56, 140.38, 146.46, 153.97, 165.97, 179.26; ^{19}F NMR (376 MHz, $CDCl_3$) δ = -147.18 – -146.80 (m); UV/Vis: λ (nm) = 495, 355, 305, 241; IR (KBr): $\tilde{\nu}$ = 3651, 3434, 2929, 2864, 1737,

1709, 1631, 1551, 1510, 1475, 1440, 1409, 1372, 1308, 1225, 1203, 1161, 1114, 1081, 1028, 986, 824, 715, 503, 482 cm^{-1}; HRMS (ESI) $C_{20}H_{27}BF_2N_2NaO_2$ calcd: 399.2026 [M+Na$^+$], found: 399.2023.

7-(5,5-Difluoro-1,3,7,9-tetramethyl-5H-4λ^4,5λ^4-dipyrrolo [1,2-c:2',1'-f] [1,3,2] diazaborinin-10-yl)-N-(3,4-dihydroxybenzyl)heptanamide (**10c**)

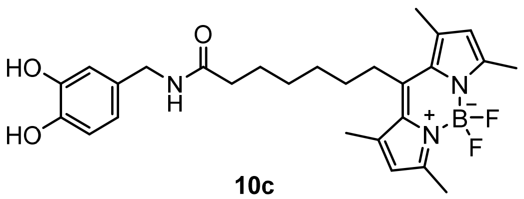

10c

10c was produced from **10b** (0.15 g, 0.4 mmol) and 4-(aminomethyl)benzene-1,2-diol hydrobromide (0.088 g, 0.4 mmol) according to Method 3. The crude material was purified by column chromatography in hexane / ethyl acetate / methanol (1:1:0.1) to afford **10c** as an orange solid (0.153 g, 77%); Melting point: 171-173 °C; R_f = 0.43 (hexane/ethyl acetate/methanol 10:10:1); ^{1}H NMR (400 MHz, DMSO-*d*$_6$) δ = 1.26 – 1.40 (m, 2H), 1.39 – 1.77 (m, 6H), 2.10 (t, *J*=7.3, 2H), 2.40 (s, 12H), 2.83 – 3.03 (m, 2H), 4.06 (d, *J*=5.8, 2H), 6.23 (s, 2H), 6.47 (dd, *J*=8.0, 2.0, 1H), 6.63 (dd, *J*=5.1, 2.9, 2H), 8.11 (t, *J*=5.8, 1H), 8.77 (s, 2H); ^{13}C NMR (101 MHz, DMSO-*d*$_6$) δ = 14.07, 15.85, 25.26, 27.75, 28.32, 29.47, 31.29, 35.27, 41.67, 114.93, 115.20, 118.13, 121.65, 130.51, 130.70, 140.75, 144.06, 145.05, 146.85, 152.98, 171.72; ^{19}F NMR (376 MHz, DMSO-*d*$_6$) δ = -145.62 – -142.53 (m); UV/Vis: λ (nm) = 495, 358, 305, 291, 236, 203; IR (KBr): $\tilde{\nu}$ = 3424, 2926, 2860, 1639, 1550, 1510, 1474, 1445, 1409, 1371, 1308, 1289, 1274, 1226, 1202, 1161, 1115, 1080, 1026, 985, 811, 714, 582, 482 cm^{-1}; HRMS (ESI) $C_{27}H_{34}BF_2N_3NaO_3$ calcd: 520.2553 [M+Na$^+$], found: 520.2554.

Tetrabenzyl (4-((7-(5,5-difluoro-1,3,7,9-tetramethyl-5H-4λ^4,5λ^4-dipyrrolo[1,2-c:2',1'-f][1,3,2]diazaborinin-10-yl)heptanamido)methyl)-1,2-phenylene) bis(phosphate) (**10d**)

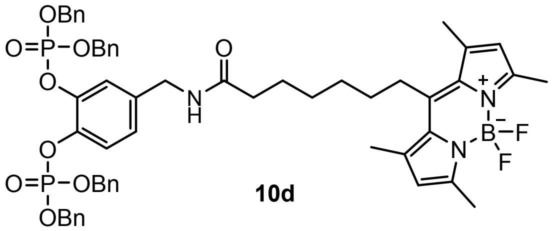

10d

10d was produced from **10c** (90 mg, 1.8 mmol) according to Method 1. The crude product was purified by column chromatography (hexane / ethyl acetate 4:1 → hexane / ethyl acetate / methanol/ acetic acid (1:1:0.1:0.01) to afford **10d** as a dark orange oil (157mg, 86%); R_f = 0.33 (hexane/ethyl acetate/MeOH/acetic acid 100:100:10:1); ^{1}H NMR (300 MHz, DMSO-*d*$_6$) δ = 1.30 (dt, *J*=8.2, 5.2, 2H), 1.39 – 1.60 (m, 6H), 2.11 (t, *J*=7.3, 2H), 2.37 (s, 12H), 2.88 (dd, *J*=10.9, 5.5, 2H), 4.20 (d, *J*=5.9, 2H), 4.96 – 5.23 (m, 8H), 6.19 (s, 2H), 7.07 (dd, *J*=8.4, 1.9, 1H), 7.18 – 7.46 (m, 21H), 8.35 (t, *J*=6.0, 1H); ^{13}C NMR (100 MHz, DMSO-*d*$_6$) δ = 14.53, 16.28, 25.66, 28.22, 28.82, 29.95, 31.66, 35.74, 41.65, 70.01 (dd, *J*=5.5, 2.3), 120.55, 121.73, 122.10, 124.96, 128.34 (d, *J*=2.2), 128.93, 128.96, 131.20, 135.88 (dd, *J*=7.0, 2.5), 138.63, 140.06 (t, *J*=6.4), 141.19, 141.27, 141.33, 147.27, 153.43, 172.67; ^{19}F NMR (282 MHz, DMSO-*d*$_6$) δ = -

144.42 (dd, J=64.5, 29.9); ^{31}P NMR (162 MHz, DMSO-d_6) δ = -5.34 (s, 1P), -5.15 (s, 1P); HRMS (ESI) $C_{55}H_{60}BF_2N_3NaO_9P_2$ calcd: 1040.3758 [M+Na$^+$], found: 1040.3786.

4-((7-(5,5-difluoro-1,3,7,9-tetramethyl-5H-4λ^4,5λ^4-dipyrrolo[1,2-c:2',1'-f][1,3,2]diazaborinin-10-yl)heptanamido)methyl)-1,2-phenylene bis(dihydrogen phosphate) (**10**)

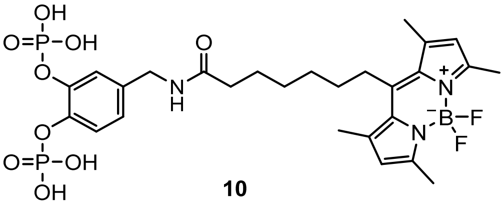

10

10 was produced from **10d** (55 mg, 0.054 mmol) according to Method 2 as a dark orange fluffy solid (35 mg, 87%); Decomposition point: >250 °C charred without melting; ^{1}H NMR (400 MHz, DMSO-d_6) δ = 1.31 – 1.38 (m, 2H), 1.45 – 1.59 (m, 6H), 2.13 (t, J=7.4, 2H), 2.40 (d, J=5.9, 12H), 2.89 – 2.99 (m, 2H), 3.92 (bs, P(O)OH + H_2O), 4.17 (d, J=5.7, 2H), 6.23 (s, 2H), 6.91 (d, J=8.0, 1H), 7.07 – 7.22 (m, 2H), 8.28 (t, 1H); ^{13}C NMR (101 MHz, DMSO-d_6) δ = 14.05, 15.85, 25.16, 27.76, 28.36, 29.43, 31.27, 35.24, 41.38, 122.43, 122.76, 130.69, 135.74, 140.76, 142.09 – 142.43 (m), 143.16 – 143.45 (m), 146.84, 152.96, 171.98; ^{31}P NMR (162 MHz, DMSO-d_6) δ = -4.33 (s, 1P), -4.17 (s, 1P); ^{19}F NMR (376 MHz, DMSO-d_6) δ = -144.22 (dd, J=61.9, 27.8); UV/Vis: λ (nm) = 529, 492, 339, 306, 276, 207, 202; IR (KBr): $\tilde{\nu}$ = 3442, 2927, 2857, 1640, 1551, 1509, 1431, 1383, 1305, 1284, 1200, 1160, 1084, 1023, 986, 825, 714, 620, 598, 581, 554, 538, 526, 516, 510, 498, 483, 470, 417 cm^{-1}; HRMS (ESI) $C_{27}H_{35}BF_2N_3O_9P_2$ calcd: 656.1915 [M-H$^+$], found: 656.1923.

N-(3,4-Dihydroxybenzyl)-2-(naphthalen-1-yloxy)acetamide (11a) and tetrabenzyl (4-((2-(naphthalen-1-yloxy)acetamido)methyl)-1,2-phenylene) bis(phosphate) (**11b**)

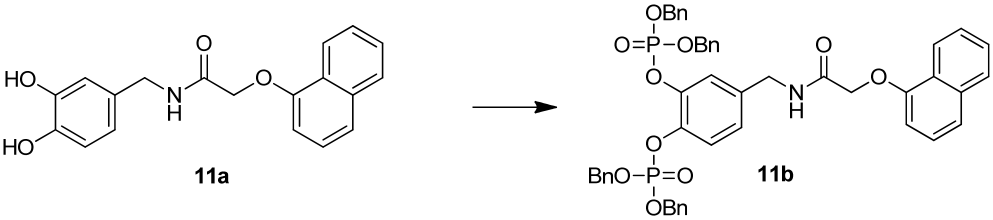

11a **11b**

11a was produced from 1-naphthoxyacetic acid (70 mg, 0.34 mmol) and 4-(aminomethyl)benzene-1,2-diol (76 mg, 0.35 mmol) according to Method 3. The crude product (97 mg) was converted according to Method 1. After purification by column chromatography in DCM/MeOH (100:1), **11b** was obtained as a colorless oil (258 mg, 90%). R_f = 0.38 (DCM/MeOH 100:1); ^{1}H NMR (400 MHz, $CDCl_3$) δ = 4.42 (d, J=6.1, 2H), 4.71 (s, 2H), 5.05 (dd, J=8.1, 2.2, 8H), 6.78 (dd, 2H), 6.87 (t, J=5.9, 1H), 6.97 (dd, J=8.4, 1.5, 1H), 7.13 – 7.31 (m, 21H), 7.31 – 7.41 (m, 1H), 7.42 – 7.55 (m, 3H), 7.69 – 7.88 (m, 1H), 8.04 – 8.22 (m, 1H); ^{13}C NMR (101 MHz, $CDCl_3$) δ = 42.21, 68.04, 70.29 (dd, J=5.8, 3.5), 105.99, 121.04 (d, J=2.4), 121.99 (d, J=2.4), 122.00, 122.14, 124.84, 125.28, 125.84, 126.02, 126.89, 127.92, 128.12 (d, J=2.2), 128.66, 128.67, 128.72, 134.74, 135.39, 135.46, 135.90, 140.84 (t, J=6.5), 141.47 (t, J=6.6), 153.00, 168.48; ^{31}P NMR (162 MHz, $CDCl_3$) δ = -5.29 (s), -5.20 (s, 1P); HRMS (ESI) $C_{47}H_{43}NaNO_{10}P_2$ calcd: 866.2254 [M+Na$^+$], found: 866.2251.

4-((2-(Naphthalen-1-yloxy)acetamido)methyl)-1,2-phenylene bis(phosphate), tetra sodium salt (**11**)

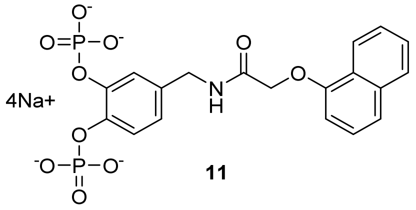

11

11b (200 mg, 0.23 mmol) was debenzylated according to Method 2. Phosphoric acid ester was obtained as as white powder (110 mg, 96%). It was dissolved in 3 ml H_2O, and an aqueous solution of $NaHCO_3$ (36 mg, 4 eq.) was added. The clear solution was filtered through cotton (4 – 6 cm in a glass pipette). The cotton was washed with 3 to 5 mL of H_2O. After lyophilization, the product was obtained as an off-white solid (125 mg, 96%). Decomposition point: >250 °C charred without melting. ^1H NMR (400 MHz, D_2O) δ = 4.30 (s, 2H), 4.75 (s, 2H), 6.71 (dd, *J*=8.4, 2.0, 1H), 6.82 (d, *J*=7.6, 1H), 7.19 – 7.31 (m, 2H), 7.36 (t, *J*=8.0, 1H), 7.42 – 7.60 (m, 3H), 7.74 – 7.90 (m, 1H), 8.13 – 8.29 (m, 1H); ^{13}C NMR (101 MHz, D_2O) δ = 42.71, 67.26, 106.18, 119.79 (d, *J*=2.4), 120.46 (d, *J*=2.3), 120.75, 121.63, 121.68, 124.95, 126.09, 126.31, 127.10, 127.77, 131.25, 134.40, 143.93 (t, *J*=5.9), 144.56 (t, *J*=6.2), 152.96, 171.29; ^{31}P NMR (162 MHz, D_2O) δ = 1.50 (s); UV/Vis: λ (nm) = 278, 220; IR (KBr): $\tilde{\nu}$ = 3417, 1661, 1598, 1582, 1542, 1509, 1463, 1427, 1398, 1385, 1353, 1282, 1269, 1240, 1228, 1109, 991, 958, 878, 834, 812, 795, 771, 703, 657, 571, 539, 530, 503, 496, 457, 443, 429 cm^{-1}; HRMS (ESI) $C_{19}H_{18}NO_{10}P_2$ calcd: 482.0411 [M^{4-}+$3H^+$], found: 482.0410.

N-(3,4-Dihydroxybenzyl)-2-(naphthalen-2-yloxy)acetamide (**12a**)

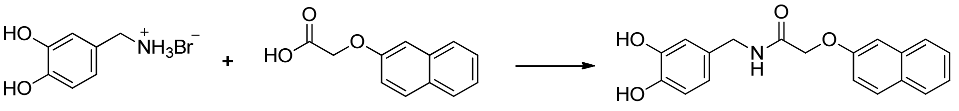

12a

12a was produced from 2-naphthoxyacetic acid (120 mg, 0.59 mmol) and 4-(aminomethyl)benzene-1,2-diol (120 mg, 0.59 mmol) according to Method 3. After purification by column chromatography in DCM/MeOH (100:1), **12a** was obtained as a white solid (163 mg, 85%); R_f = 0.16 (DCM/MeOH 100:1); ^1H NMR (400 MHz, DMSO-d_6) δ = 4.18 (d, *J*=5.8, 2H), 4.62 (s, 2H), 6.50 (dd, *J*=8.0, 2.1, 1H), 6.61 (d, *J*=8.0, 1H), 6.69 (d, *J*=2.1, 1H), 7.22 – 7.31 (m, 2H), 7.35 (ddd, *J*=8.1, 6.8, 1.2, 1H), 7.45 (ddd, *J*=8.2, 6.9, 1.3, 1H), 7.76 (dd, *J*=8.2, 1.2, 1H), 7.80 – 7.87 (m, 2H), 8.54 (t, *J*=6.2, 1H), 8.76 (s, 2H); ^{13}C NMR (75 MHz, DMSO-d_6) δ = 41.52, 67.04, 107.27, 115.02, 115.25, 118.25, 118.68, 123.84, 126.46, 126.74, 127.50, 128.73, 129.31, 130.10, 134.03, 144.16, 145.06, 155.59, 167.25; MS (ESI) $C_{19}H_{16}NO_4$ calcd: 322.1 [M-H^+], found: 322.0.

Tetrabenzyl (4-((2-(naphthalen-2-yloxy)acetamido)methyl)-1,2-phenylene) bis(phosphate) (**12b**)

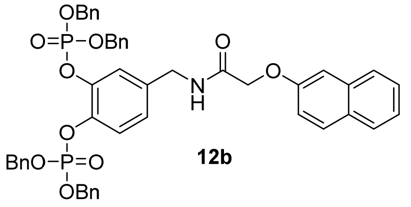

12b

12b was produced from **12a** (120 mg, 0.37 mmol) according to Method 1. After purification by column chromatography (DCM/MeOH 100:1), 12b was obtained as a colorless oil (280 mg, 90%); R_f = 0.20 (DCM/MeOH 100:1); ^{1}H NMR (300 MHz, DMSO-d_6) δ = 4.32 (d, *J*=6.1, 2H), 4.65 (s, 2H), 4.94 – 5.24 (bs, 8H), 7.10 (dd, *J*=8.9, 1.3, 1H), 7.21 – 7.38 (m, 25H), 7.43 (ddd, *J*=8.2, 6.9, 1.4, 1H), 7.69 – 7.92 (m, 3H), 8.80 (t, *J*=6.2, 1H); ^{13}C NMR (101 MHz, DMSO-d_6) δ = 41.09, 67.03, δ = 69.54 (t, *J*=5.3), 107.33, 118.63, 120.33 – 120.40 (m), 121.22, 123.87, 124.63, 126.46, 126.74, 127.49, 127.86, 128.00, 128.45, 128.48, 128.75, 129.34, 134.01, 135.41 (d, *J*=7.0, 1.0), 137.62, 139.69 (t, *J*=6.4), 140.71 (t, *J*=6.5), 155.50, 167.76; ^{31}P NMR (162 MHz, DMSO-d_6) δ = -5.31 (s, 1P), -5.16 (s, 1P); MS (ESI) $C_{47}H_{43}NaNO_{10}P_2$ calcd: 866.2254 [M+Na$^+$], found: 866.2250.

4-((2-(Naphthalen-2-yloxy)acetamido)methyl)-1,2-phenylene bis(dihydrogen phosphate) (**12**)

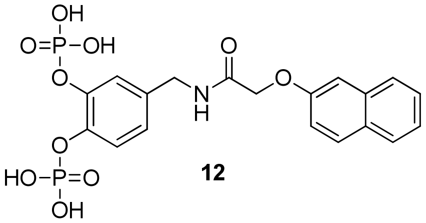

12

12 was produced from **12b** (200 mg, 0.24 mmol) according to Method 2 as a solid (105 mg, 92%); Melting point: 73-75 °C; ^{1}H NMR (400 MHz, DMSO-d_6) δ = 4.29 (d, *J*=5.8, 2H), 4.65 (s, 2H), 5.15 – 6.47 (bs, POH + H_2O), 6.90 – 7.02 (m, 1H), 7.08 – 7.39 (m, 4H), 7.45 (t, *J*=7.5, 1H), 7.68 – 7.95 (m, 3H), 8.73 (d, *J*=6.2, 1H); ^{13}C NMR (101 MHz, DMSO-d_6) δ = 41.31, 67.03, 107.32, 118.69, 121.56, 122.25, 123.13, 123.88, 126.50, 126.80, 127.52, 128.76, 129.36, 134.05, 135.59, 155.59, 167.61; ^{31}P NMR (162 MHz, DMSO-d_6) δ = -4.59 (s, P), -4.44 (s, 1P); UV/Vis: λ (nm) = 325, 311, 270, 226; IR (KBr): $\tilde{\nu}$ = 3409, 3558, 2924, 2853, 2715, 2332, 1648, 1630, 1600, 1552, 1510, 1468, 1429, 1392, 1350, 1270, 1258, 1218, 1182, 1157 , 1122, 1017, 980, 915, 839, 813, 748, 726, 708, 622, 580, 505, 497, 472, 426 cm^{-1}; HRMS (ESI) $C_{19}H_{18}NO_{10}P_2$ calcd: 482.0411 [M-H$^+$], found: 482.0410.

Benzyl 6-hydroxy-2-naphthoate (**13a**)

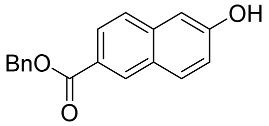

13a

$KHCO_3$ (0.32 g, 3.20 mmol) and benzyl bromide (0.38 ml, 3.20 mmol) were added to a solution of 6-hydroxy-2-naphthoic acid (0.5 g, 2.66 mmol) in 10 mL dry DMF and stirred at 40 °C for 6 hr. Upon completion of reaction, 100 mL of water was added and the product was extracted with ethyl acetate (3 x 50 mL). The organic layer was washed with 5% $NaHCO_3$ and dried over Na_2SO_4. Volatiles were removed under reduced pressure. The crude product was purified by column chromatography (hexane / ethyl acetate 9:1 to 4:1) to afford **13a** as off-white solid (0.72 g, 99.9%); Melting point: 119 – 121 °C; R_f = 0.35 (hexane:ethyl acetate 4:1); ^{1}H NMR (400 MHz, DMSO-d_6) δ = 5.37 (s, 2H), 7.15 (dd, *J*=11.7, 3.0, 2H), 7.29 – 7.45 (m, 3H), 7.45 – 7.53 (m, 2H), 7.76 (d, *J*=8.7, 1H), 7.87 (dd, *J*=8.6, 1.7, 1H), 7.97 (d, *J*=8.8, 1H), 8.51 (s, 1H), 10.17 (s, 1H); ^{13}C NMR (75 MHz, DMSO-d_6) δ = 65.97, 108.68, 119.64, 123.55, 125.10, 126.43, 126.56, 127.96, 128.04, 128.50, 130.60, 131.32, 136.31, 137.16, 157.79, 165.85; UV/Vis: λ (nm) = 304, 253, 246, 239; IR (KBr): $\tilde{\nu}$ = 3564, 3542, 3520, 3359, 3066, 3029, 1687, 1674, 1620, 1603, 1578, 1498, 1480, 1454, 1445, 1436, 1394, 1378, 1353, 1314, 1295, 1282, 1204, 1160, 1128, 1099, 1031, 1001, 989, 971, 959, 955, 942, 910, 883, 864, 828, 817, 767, 752, 738, 696, 659, 651, 621, 604, 590, 540, 527, 514, 472, 457 cm^{-1}; MS (ESI) $C_{18}H_{14}NaO_3$ calcd: 301.1 [M+Na$^+$], found: 301.1.

Benzyl 6-(2-ethoxy-2-oxoethoxy)-2-naphthoate (**13b**)

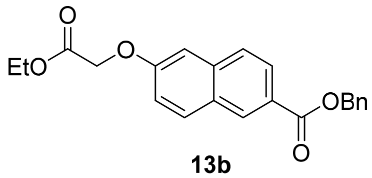

13b

Powdered and dried K_2CO_3 (0.5 g, 3.6 mmol) and ethyl bromoacetate (0.24 ml, 2.16 mmol) were added to the solution of **13a** (0.5 g, 1.8 mmol) in dry DMF (6 ml), and the mixture was stirred at ambient conditions for 1 h. After addition of 100 mL of water, the product was extracted with ethyl acetate (3 x 50 mL). The combined organic layer was dried over Na_2SO_4. Volatiles were removed under reduced pressure to afford **13b** as white solid (0.65 g, 99%); Melting point: 76 – 78 °C; ^{1}H NMR (300 MHz, $CDCl_3$) δ = 1.31 (t, *J*=7.1, 3H), 4.30 (q, *J*=7.1, 2H), 4.75 (s, 2H), 5.42 (s, 2H), 7.10 (d, *J*=2.4, 1H), 7.23 – 7.31 (m, 1H), 7.32 – 7.45 (m, 3H), 7.45 – 7.53 (m, 2H), 7.74 (d, *J*=8.6, 1H), 7.87 (d, *J*=9.0, 1H), 8.07 (dd, *J*=8.6, 1.7, 1H), 8.57 (s, 1H); UV/Vis: λ (nm) = 299, 253, 247, 240; IR (KBr): $\tilde{\nu}$ = IR: 3626, 3584, 3564, 3543, 3523, 3489, 3458, 3445, 3394, 3066, 3038, 2980, 2962, 2938, 2907, 1754, 1705, 1666, 1630, 1607, 1588, 1501, 1480, 1453, 1434, 1421, 1491, 1341, 1304, 1284, 1234, 1204, 1182, 1135, 1106, 1071, 1033, 1000, 966, 937, 923, 865, 813, 769, 750, 740, 699, 597, 585, 540, 470, 421 cm^{-1}; MS (ESI) $C_{22}H_{20}NaO_5$ calcd: 387.1 [M+Na$^+$], found: 387.2.

6-(2-Ethoxy-2-oxoethoxy)-2-naphthoic acid (**13c**)

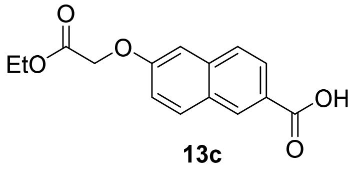

13c

To the solution of **13b** (0.6 g, 1.6 mmol) in ethyl acetate / ethanol (1:1) was added Pd/C (0.06 g) under an argon atmosphere. The argon atmosphere was exchanged for a hydrogen

atmosphere. After 3 h, the mixture is filtered through celite, and was washed with ethanol. After evaporation of the solvent under reduced pressure, **13c** was obtained as white off-white solid (0.45 g, 99.9%); Melting point: 182 – 184 °C; ^{1}H NMR (300 MHz, DMSO-d_6) δ = 1.21 (t, *J*=7.1, 3H), 4.18 (q, *J*=7.1, 2H), 4.92 (s, 2H), 7.28 (dd, *J*=9.0, 2.6, 1H), 7.35 (d, *J*=2.4, 1H), 7.83 (d, *J*=8.7, 1H), 7.92 (dd, *J*=8.6, 1.6, 1H), 8.03 (d, *J*=9.0, 1H), 8.51 (s, 1H); ^{13}C NMR (75 MHz, DMSO-d_6) δ = 14.04, 60.73, 64.76, 107.19, 119.15, 125.89, 126.37, 126.95, 127.77, 130.27, 131.03, 136.32, 157.25, 160.61, 167.53, 168.41; UV/Vis: λ (nm) = 293, 242, 236, 214; IR (KBr): $\tilde{\nu}$ = 3647, 3583, 3564, 3544, 3523, 3501, 3484, 3463, 3448, 3062, 2980, 2909, 2868, 2676, 2629, 2561, 1766, 1693, 1630, 1580, 1504, 1482, 1441, 1415, 1389, 1341, 1301, 1261, 1201, 1174, 1131, 1081, 1028, 928, 913, 852, 822, 766, 749, 698, 604, 562, 471 cm^{-1}; HRMS (ESI) $C_{15}H_{13}NO_5$ calcd: 273.0768 [M-H$^+$], found: 273.0771.

Ethyl 2-((6-(phenylcarbamoyl)naphthalen-2-yl)oxy)acetate (**13d**)

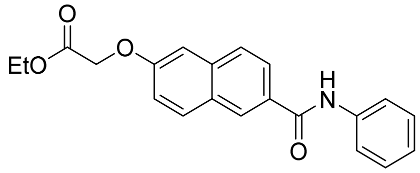

13d

13d was produced from **13c** (0.5 g, 1.8 mmol) and aniline (0.2 g, 2.73 mmol) in dry DMF according to Method 3. The crude product was purified by column chromatography in DCM / acetone (20:1) to yield **13d** as an off-white solid (0.6 g, 94%); Melting point: 154 – 156 °C; R_f = 0.34 (DCM/acetone 20:1); ^{1}H NMR (300 MHz, $CDCl_3$) δ = 1.32 (t, *J*=7.1, 3H), 4.31 (q, *J*=7.1, 2H), 4.76 (s, 2H), 7.10 (d, *J*=2.5, 1H), 7.12 – 7.22 (m, 1H), 7.30 (dd, *J*=9.0, 2.6, 1H), 7.39 (t, *J*=7.9, 2H), 7.63 – 7.74 (m, 2H), 7.75 – 7.94 (m, 3H), 7.98 (s, 1H), 8.30 (s, 1H); ^{13}C NMR (75 MHz, $CDCl_3$) δ = 14.32, 61.71, 65.53, 107.08, 119.89, 120.36, 124.46, 124.64, 127.48, 127.68, 128.59, 129.24, 130.59, 130.97, 136.19, 138.21, 157.49, 165.85, 168.66; UV/Vis: λ (nm) = 279, 248, 240; IR (KBr): $\tilde{\nu}$ = 3646, 3564, 3545, 3523, 3499, 3447, 3262, 3195, 3136, 3065, 2983, 2924, 1758, 1717, 1641, 1602, 1547, 1501, 1481, 1442, 1392, 1335, 1305, 1276, 1241, 1203, 1172, 1132, 1098, 1077, 1021, 965, 952, 912, 892, 862, 844, 821, 752, 716, 694, 601, 593, 533, 507, 475 cm^{-1}; HRMS (ESI) $C_{21}H_{20}NO_4$ calcd: 350.1387 [M+H$^+$], found: 350.1385.

2-((6-(Phenylcarbamoyl) naphthalen-2-yl)oxy)acetic acid (**13e**)

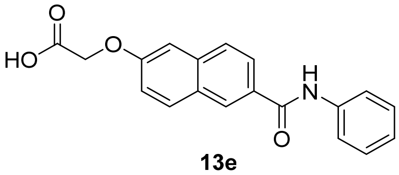

13e

To a solution of **13d** (0.55 g, 1.57 mmol) in THF (5 ml), an aqueous solution of 1 M NaOH (5 mL) was added dropwise, and the reaction was stirred at room temperature for 1 h. The reaction was diluted with 50 mL water and acidified to pH 1-2 by addition of 1 N HCl. After extraction of the product with ethyl acetate (3 x 50 ml) and removal of the solvent under reduced pressure, **13e** was obtained as a white solid (0.5 g, 99%); Melting point: 212 – 215 °C; ^{1}H NMR (400 MHz, DMSO-d_6) δ = 4.83 (s, 2H), 7.09 (tt, *J*=7.4, 1.2, 1H), 7.29 (dd, *J*=8.9, 2.5, 1H), 7.32 – 7.40 (m, 3H), 7.76 – 7.85 (m, 2H), 7.90 (d, *J*=8.7, 1H), 7.95 – 8.04 (m, 2H), 8.50 (d, *J*=1.9, 1H), 10.32 (s, 1H), 13.05 (s, 1H); ^{13}C NMR (75 MHz, DMSO-d_6) δ = 64.60, 106.98, 119.34, 120.33, 123.54, 125.02, 126.83, 127.59, 127.83, 128.59, 130.12, 130.64,

135.68, 139.30, 157.09, 160.61, 165.53, 169.91; UV/Vis: λ (nm) = 299, 239, 210, 204; IR (KBr): $\tilde{\nu}$ = 3649, 3628, 3607, 3586, 3566, 3545, 3524, 3502, 3481, 3462, 3446, 3343, 3059, 3038, 2924, 2909, 2555, 1764, 1745, 1718, 1685, 1648, 1628, 1598, 1534, 1505, 1480, 1442, 1411, 1392, 1346, 1321, 1273, 1238, 1205, 1173, 1134, 1079, 904, 883, 856, 847, 819, 749, 688, 660, 589, 502, 476 cm^{-1}; HRMS (ESI) $C_{19}H_{14}NO_4$ calcd: 320.0928 [M-H^+], found: 320.0929.

6-(2-((3,4-Dihydroxybenzyl)amino)-2-oxoethoxy)-N-phenyl-2-naphthamide (**14**)

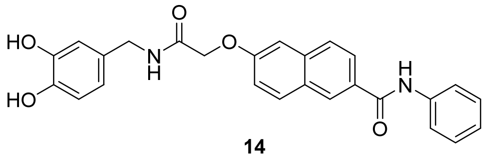

14

14 was produced from **13e** (0.36 g, 1.12 mmol) and 4-(aminomethyl)benzene-1,2-diol (0.27 g, 1.23 mmol) according to Method 3. The crude product was purified by column chromatography in DCM/MeOH (50:1) to afford the product as a white solid (0.35 g, 70 %); Melting point: 219 – 222 °C; R_f = 0.43 (DCM/MeOH 50:1); ^{1}H NMR (400 MHz, DMSO-d_6) δ = 4.18 (d, *J*=6.0, 2H), 4.67 (s, 2H), 6.51 (dd, *J*=8.0, 2.1, 1H), 6.62 (d, *J*=8.0, 1H), 6.70 (d, *J*=2.1, 1H), 7.03 – 7.15 (m, 1H), 7.30 – 7.40 (m, 4H), 7.80 (dd, *J*=8.6, 1.1, 2H), 7.88 (d, *J*=8.7, 1H), 7.95 – 8.06 (m, 2H), 8.50 (d, *J*=1.5, 1H), 8.58 (t, *J*=6.1, 1H), 8.71 (s, 1H), 8.80 (s, 1H), 10.32 (s, 1H); ^{13}C NMR (101 MHz, DMSO-d_6) δ = 41.53, 67.04, 107.20, 115.02, 115.24, 118.25, 119.60, 120.32, 123.55, 125.04, 126.85, 127.66, 127.84, 128.59, 130.06, 130.20, 130.57, 135.66, 139.30, 144.16, 145.06, 157.07, 165.55, 167.07; UV/Vis: λ (nm) = 285, 237, 204; IR (KBr): $\tilde{\nu}$ = 3839, 3820, 3802, 3748, 3737, 3673, 3647, 3564, 3417, 2324, 1746, 1736, 1715, 1651, 1630, 1603, 1535, 1506, 1478, 1442, 1393, 1363, 1323, 1273, 1239, 1203, 1173, 1117, 1066, 1022, 948, 906, 885, 858, 812, 756, 691, 631, 587, 505, 474, 421 cm^{-1}; HRMS (ESI) $C_{26}H_{21}N_2O_5$ calcd: 441.1456 [M-H^+], found: 441.1457.

Tetrabenzyl (4-((2-((6-(phenylcarbamoyl) naphthalen-2-yl) oxy) acetamido)methyl)-1,2-phenylene) bis(phosphate) (**13f**)

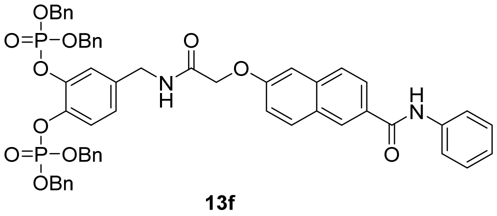

13f

13f was produced from **14** (0.28 g, 0.63 mmol) according to Method 1. The crude product was purified by column chromatography (DCM/acetone 20:1 → 9:1) to yield **13f** as a viscous oil (0.485 g, 80%); R_f = 0.26 (DCM/acetone 9:1); ^{1}H NMR (400 MHz, $CDCl_3$) δ = 4.35 (d, *J*=6.1, 2H), 4.55 (s, 2H), 4.96 – 5.10 (m, 8H), 6.83 (dd, *J*=8.5, 1.6, 1H), 7.00 (d, *J*=2.2, 1H), 7.07 – 7.34 (m, 27H), 7.61 (dd, *J*=23.8, 8.8, 2H), 7.78 (d, *J*=7.3, 2H), 7.88 (dd, *J*=8.6, 1.8, 1H), 8.27 (d, *J*=1.8, 1H), 9.02 – 9.14 (m, 1H); ^{13}C NMR (101 MHz, $CDCl_3$) δ = 41.95, 67.24, 70.11 – 70.28 (m), 107.38, 118.97, 120.44, 121.01 (d, *J*=2.2), 121.59 (d, *J*=2.0), 124.16, 124.84, 125.00, 127.19, 127.79, 127.94 (d, *J*=2.1), 128.42, 128.57 (d, *J*=1.3), 128.66, 128.87, 130.99, 131.05, 135.13 (dd, *J*=7.0, 4.1), 135.76, 135.96, 138.73, 140.52 (t, *J*=6.3), 141.09 (t, *J*=6.7),

156.26, 166.33, 167.99; ^{31}P NMR (162 MHz, $CDCl_3$) δ = -5.53 (s, 1P), -5.44 (s, 1P); UV/Vis: λ (nm) = 280, 240; IR (KBr): $\tilde{\nu}$ = 3645, 3633, 3625, 3604, 3592, 3583, 3572, 3563, 3426, 3308, 3062, 3023, 2924, 2897, 1768, 1667, 1660, 1652, 1629, 1600, 1539, 1506, 1478, 1456, 1441, 1393, 1321, 1274, 1203, 1173, 1157, 1124, 1081, 1018, 1001, 880, 813, 741, 695, 596, 511, 499, 475, 456 cm^{-1}; HRMS (ESI) $C_{54}H_{48}N_2N_aO_{11}P_2$ calcd: 985.2626 [M+Na$^+$], found: 985.2621.

4-((2-((6-(phenylcarbamoyl) naphthalen-2-yl)oxy) acetamido)methyl)-1,2-phenylene bis(dihydrogen phosphate) (**13**)

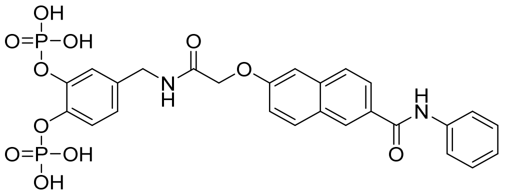

13

13 was produced from **13f** (0.3 g, 0.31 mmol) according to Method 2**.** Lyophilization yielded **13** as a white solid (0.178 g, 95%). Melting point: 192 – 194 °C; ^{1}H NMR (400 MHz, DMSO-d_6) δ = 4.30 (d, *J*=6.0, 2H), 4.70 (s, 2H), 6.99 (d, *J*=8.2, 1H), 7.09 (t, *J*=7.4, 1H), 7.21 (d, *J*=8.3, 1H), 7.26 (s, 1H), 7.30 – 7.43 (m, 4H), 7.80 (d, *J*=7.7, 2H), 7.90 (d, *J*=8.6, 1H), 7.94 – 8.07 (m, 2H), 8.50 (s, 1H), 8.76 (t, *J*=6.1, 1H), 10.34 (s, 1H); ^{13}C NMR (101 MHz, DMSO-d_6) δ = 41.29, 67.01, 107.26, 119.59, 120.34, 121.32, 122.02, 123.21, 123.56, 125.08, 126.90, 127.69, 127.85, 128.61, 130.25, 130.62, 135.67, 139.32, 141.68, 142.61, 157.05, 165.60, 167.43; ^{31}P NMR (162 MHz, DMSO-d_6) δ = -4.66 (s, 1P), -4.50 (s, 1P); UV/Vis: λ (nm) = 301, 250, 238, 201; IR (KBr): $\tilde{\nu}$ = 3434, 1651, 1631, 1601, 1537, 1510, 1479, 1441, 1394, 1324, 1273, 1236, 1208, 1175, 1122, 1076, 1065, 1020, 977, 951, 914, 842, 823, 753, 692 ,584, 569, 558, 539, 526, 512, 502, 495, 481, 473 cm^{-1}; HRMS (ESI) $C_{26}H_{23}NO_{11}P_2$ calcd: 601.0783 [M-H$^+$], found: 601.0788.

Dibenzyl (4-((2-(naphthalen-2-yloxy)acetamido)methyl)phenyl) phosphate (**15b**)

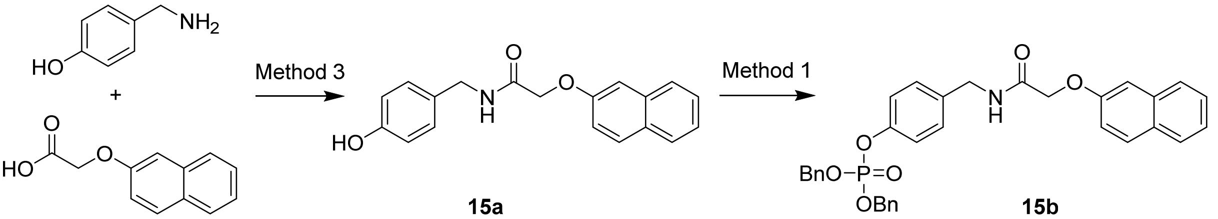

15a **15b**

15a was prepared from 2-naphthoxy acetic acid (0.25 g, 1.2 mmol) and 4-hydroxybenzylamine (0.23 g, 1.86 mmol) according to Method 3. The crude **15a** was used for the following step without purification according to Method 1. After column chromatography (DCM/acetone 20:1 → 9:1), **15b** was obtained as an oil (0.25 g, 37 % over 2 steps). Melting point: 84 – 86 °C; R_f = 0.29 (DCM/acetone 9:1); ^{1}H NMR (400 MHz, $CDCl_3$) δ = 4.49 (d, *J*=6.0, 2H), 4.63 (s, 2H), 5.10 (d, *J*=8.5, 4H), 7.09 (d, *J*=8.6, 2H), 7.11 – 7.26 (m, 5H), 7.33 (dq, *J*=5.6, 3.6, 3.1, 11H), 7.45 (t, *J*=7.5, 1H), 7.73 (q, *J*=8.9, 8.3, 3H); ^{13}C NMR (101 MHz, $CDCl_3$) δ = 42.10, 67.27, 69.84 (d, *J*=5.8), 107.49, 118.03, 120.12 (d, *J*=4.8), 124.23, 126.62, 126.84, 127.54, 127.88, 128.47, 128.53, 128.96, 129.32, 129.74, 134.11, 134.80, 135.24 (d, *J*=6.8), 149.72 (d, *J*=6.9),

154.90, 167.97; ^{31}P NMR (162 MHz, $CDCl_3$) δ = -5.06; HRMS (ESI) $C_{33}H_{30}NNaO_6P$ calcd: 590.1703 [M+Na$^+$], found: 590.1701.

4-((2-(naphthalen-2-yloxy)acetamido)methyl)phenyl dihydrogen phosphate (**15**)

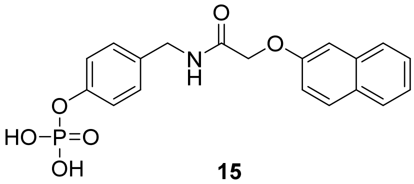

15

15 was produced from **15b** (0.2 g, 0.35 mmol) according to Method 2**.** Lyophilization yielded **15** as a white solid (0.127 g, 93%). Melting point: 195 – 197 °C; ^{1}H NMR (400 MHz, DMSO-*d*$_6$) δ = 4.32 (d, *J*=6.0, 2H), 4.67 (s, 2H), 7.07 (d, *J*=8.3, 2H), 7.21 (d, *J*=8.4, 2H), 7.24 – 7.32 (m, 2H), 7.36 (t, *J*=7.4, 1H), 7.47 (t, *J*=7.3, 1H), 7.78 (d, *J*=8.2, 1H), 7.85 (dd, *J*=8.4, 5.2, 2H), 8.70 (t, *J*=6.0, 1H); ^{13}C NMR (101 MHz, DMSO-*d*$_6$) δ = 41.31, 67.04, 107.29, 118.69, 119.83, 119.87, 123.86, 126.49, 126.73, 127.51, 128.28, 128.74, 129.33, 134.02, 134.44, 150.79 (d, *J*=6.2), 155.57, 167.55; ^{31}P NMR (162 MHz, DMSO-*d*$_6$) δ = -4.49; HRMS (ESI) $C_{19}H_{18}N_2O_6P$ Calcd: 386.0799 [M-H$^+$], found: 386.0799.

((((4-((2-((6-(phenylcarbamoyl)naphthalen-2-yl)oxy)acetamido)methyl)-1,2-phenylene)bis(oxy))bis(oxo-l5-phosphanetriyl))tetrakis(oxy))tetrakis(methylene) tetrakis(2,2-dimethylpropanoate) (**17**)

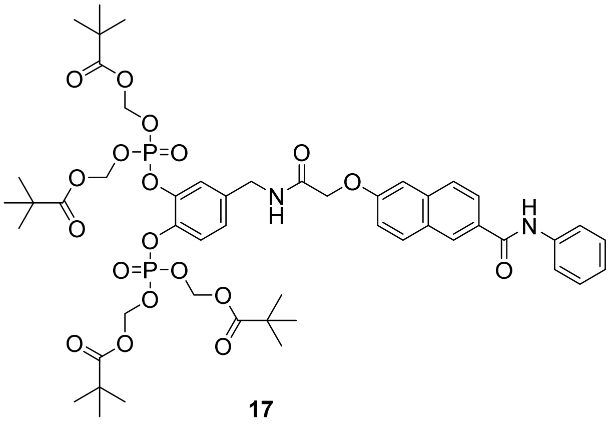

17

13 (40 mg, 0.07 mmol) was suspended in dry acetonitrile. Diisopropylethyl amine (0.1 ml, 0.55 mmol) and iodomethyl pivalate (130 mg, 0.55 mmol) were added. After stirring at room temperature overnight, the suspension had turned into a clear solution. Volatile components were removed in vacuo. The product was purified by column chromatography in (hexane / ethyl acetate 3:1 → 2:1) to yield **17** as a colorless oil (35 mg, 50%); R_f = 0.15 (hexane/acetone 2:1); ^{1}H NMR (400 MHz, $CDCl_3$-*d*) δ = 1.18 (d, *J*=4.6, 36H), 4.48 (d, *J*=6.1, 2H), 4.68 (s, 2H), 5.60 – 5.79 (m, 8H), 6.94 – 7.06 (m, 2H), 7.11 – 7.18 (m, 2H), 7.18 – 7.25 (m, 2H), 7.29 (s, 1H), 7.33 – 7.40 (m, 2H), 7.75 (dd, *J*=18.0, 7.9, 3H), 7.84 (d, *J*=9.0, 1H), 7.91 (dd, *J*=8.6, 1.8, 1H), 8.32 (s, 1H), 8.44 (d, *J*=5.4, 1H); ^{13}C NMR (101 MHz, $CDCl_3$) δ = 26.96, 38.90, 42.19, 67.56, 77.33, 83.35, 83.39, 83.41, 83.45, 107.75, 119.37, 120.46, 121.37 – 121.46 (m), 121.96 – 122.05 (m), 124.58, 124.97, 125.65, 127.70, 127.86, 128.81, 129.24, 131.30, 136.13, 136.65, 138.57, 140.30 (t, *J*=6.7), 140.86 (t, *J*=6.9), 156.60, 156.65, 166.19, 168.15, 176.72, 176.79; ^{31}P NMR (162 MHz, $CDCl_3$) δ = -8.73 (s, 1P), -9.00 (s, 1P); HRMS (ESI) $C_{50}H_{64}N_2NaO_{19}P_2$ calcd: 1081.3471 [M+Na$^+$], found: 1081.3461.

(((2-(phosphorylmethyl) benzyl)phosphoryl)tetrakis(oxy))tetrakis(methylene) tetrakis(2,2-dimethylpropanoate) (**18**)

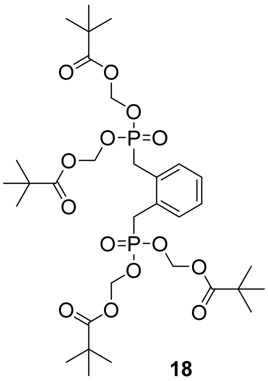

18

To the solution of **3** (100 mg, 0.37 mmol) in dry DMF were added diisopropyl ethylamine (1 mL, 6 mmol) and iodomethyl pivalate (680 mg, 4.5 mmol). After stirring for 20 h at room temperature, the reaction mixture was diluted with ethyl acetate and washed with saturated $NaHCO_3$ and brine solution. The combined organic layer was dried over Na_2SO_4. After evaporation of the solvents and purification by column chromatography (hexane / ethyl acetate 4:1 → 3:1 → 3:2), **18** was obtained as a white solid (163 mg, 60%); R_f = 0.28 (hexane/ethyl acetate 3:2); ^{1}H NMR (400 MHz, $CDCl_3$) δ = 1.19 (s, 36H), 3.44 (d, *J*=20.7, 4H), 5.50 – 5.63 (m, 8H), 7.17 – 7.29 (m, 5H); ^{13}C NMR (101 MHz, $CDCl_3$) δ = 26.95, 30.87 (d, J=2.0), 32.24 (d, J=2.1), 38.82, 76.83, 77.15, 77.47, 81.67, 127.88, 129.69 – 130.12 (m), 131.90, 176.94; ^{31}P NMR (162 MHz, $CDCl_3$) δ = 28.08 (s); HRMS (ESI) $C_{32}H_{52}NaO_{14}P_2$ calcd: 745.2725 [M+Na$^+$], found: 745.2722.

Benzyl 2-(2-hydroxyphenyl)acetate (**SI-1a**)

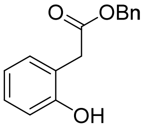

To the solution of 2-hydroxyphenylacetic acid (1 g, 6.57 mmol) in 10 mL of dry DMF was added sodium bicarbonate (0.83 g, 9.85 mmol) and benzyl bromide (1.17 ml, 9.85 mmol). After stirring for 16 h at room temperature, the mixture was diluted with water (100 mL) and extracted with ethyl acetate (3x25 mL). The combined organic layers were washed with saturated aqueous $NaHCO_3$ and brine, and were dried over Na_2SO_4. After removal of the solvents under reduced pressure, the crude material was purified by column chromatography (hexane / ethyl acetate 9:1 → 4:1) to yield **SI-1a** (1.02 g, 65%). R_f = 0.39 (hexane / ethyl acetate 4:1); ^{1}H NMR (400 MHz, $CDCl_3$) δ = 3.76 (s, 2H), 5.20 (s, 2H), 6.91 (td, *J*=7.4, 1.2, 1H), 6.96 (dd, *J*=8.1, 1.1, 1H), 7.13 (dd, *J*=7.5, 1.5, 1H), 7.22 (td, *J*=7.8, 1.7, 1H), 7.31 (s, 1H), 7.33 – 7.44 (m, 5H); ^{13}C NMR (101 MHz, $cdcl_3$) δ = 37.9, 67.7, 117.7, 120.7, 121.0, 128.5, 128.7, 128.8, 129.3, 131.2, 135.2, 155.2, 173.8; MS (ESI) $C_{15}H_{14}NaO_3$ Calcd: 265.1 [M+Na$^+$], found: 265.0.

Benzyl 2-(2-((bis(benzyloxy)phosphoryl)oxy)phenyl)acetate (**SI-1b**)

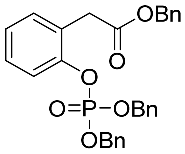

SI-1b was synthesized from **SI-1a** (0.52 g, 0.21 mmol) according to Method 1. The crude product was purified by column chromatography (hexane / ethyl acetate 9:1 → 4:1 to yield **SI-1b** (0.91 g, 84%). R_f = 0.32 (hexane / ethyl acetate 4:1); ^{1}H NMR (400 MHz, $CHCl_3$-*d*) δ = 3.70 (s, 2H), 4.98 – 5.14 (m, 6H), 7.14 (t, *J*=7.5, 1H), 7.18 – 7.35 (m, 17H), 7.38 (d, *J*=8.1, 1H); ^{31}P NMR (162 MHz, $CDCl_3$) δ = -5.2; ^{13}C NMR (101 MHz, $CDCl_3$) δ = 36.0, 66.7, 70.11 (d, *J*=5.9), 119.93 (d, *J*=2.3), 125.3, 125.59 (d, *J*=7.4), 128.1, 128.2, 128.3, 128.86 (d, *J*=1.3), 128.7, 128.9, 128.9, 131.6, 135.55 (d, *J*=7.0), 135.9, 149.15 (d, *J*=6.7), 170.8; MS (ESI) $C_{29}H_{27}NaO_6P$ calcd: 525.1 [M+Na$^+$], found: 525.1.

2-(2-(phosphonooxy)phenyl)acetic acid (**SI-1**)

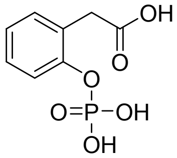

SI-1 was produced from **SI-1b** (0.06 g, 0.12 mmol) according to Method 2. Lyophilization yielded **SI-1** as a white solid (0.027 g, 99%). Melting point: 146 – 148 °C; ^{1}H NMR (400 MHz, CD_3OD) δ = 3.76 (s, 2H), 7.15 (t, *J*=7.5, 1H), 7.17 – 7.40 (m, 3H); ^{31}P NMR (162 MHz, CD_3OD) δ = -5.3; ^{13}C NMR (101 MHz, CD3OD) δ = 35.17 (d, *J*=3.2), 171.88 – 174.74 (m), 119.98 (dd, *J*=11.1, 2.3), 124.39 , 126.31 (d, *J*=6.7), 126.62 (d, *J*=6.4), 128.21 (d, *J*=12.3), 131.31 (d, *J*=8.4), 150.14 (d, *J*=6.5), 172.49 , 174.00; HRMS (ESI) $C_8H_8O_6P$ calcd: 231.0064 [M-H$^+$], found: 231.0063.

Supporting references

[1] D. Neculai, A. M. Neculai, S. Verrier, K. Straub, K. Klumpp, E. Pfitzner, S. Becker, *J. Biol. Chem.* **2005**, *280*, 40782-40787.

[2] M. Gräber, W. Janczyk, B. Sperl, N. Elumalai, C. Kozany, F. Hausch, T. A. Holak, T. Berg, *ACS Chem. Biol.* **2011**, *6*, 1008-1014.

[3] Z. Nikolovska-Coleska, R. Wang, X. Fang, *et al.*, *Anal. Biochem.* **2004**, *332*, 261-273.

[4] C. H. Reynolds, B. A. Tounge, S. D. Bembenek, *J. Med. Chem.* **2008**, *51*, 2432-2438.

[5] O. Trott, A. J. Olson, *J. Comput. Chem.* **2010**, *31*, 455-461.

[6] C. A. Schneider, W. S. Rasband, K. W. Eliceiri, *Nat. Methods* **2012**, *9*, 671-675.

[7] L. J. Silverberg, J. L. Dillon, P. Vemishetti, *Tetrahedron Lett.* **1996**, *37*, 771-774.

[8] S. J. Mills, F. Vandeput, M. N. Trusselle, S. T. Safrany, C. Erneux, B. V. Potter, *ChemBioChem* **2008**, *9*, 1757-1766.

[9] B. Suh, R. Barker, *J. Biol. Chem.* **1971**, *246*, 7041-7050.

[10] D. P. Flaherty, T. Kiyota, Y. X. Dong, T. Ikezu, J. L. Vennerstrom, *J. Med. Chem.* **2010**, *53*, 7992-7999.

[11] A. Winter, C. Friebe, M. D. Hager, U. S. Schubert, *Eur. J. Org. Chem.* **2009**, 801-809.

[12] L. Maier, M. M. Crutchfield, *Phosphorus Sulfur* **1978**, *5*, 45-51.

[13] M. B. Gawande, P. S. Branco, *Green Chem.* **2011**, *13*, 3355-3359.

[14] I. A. Boldyrev, J. G. Molotkovsky, *Russ. J. Bioorg. Chem.* **2006**, *32*, 78-83.

[15] A. B. Nepomnyashchii, M. Broring, J. Ahrens, A. J. Bard, *J. Am. Chem. Soc.* **2011**, *133*, 8633-8645.
